# Immune Responses to Multi-Frequencies of 1.5 GHz and 4.3 GHz Microwave Exposure in Rats: Transcriptomic and Proteomic Analysis

**DOI:** 10.3390/ijms23136949

**Published:** 2022-06-22

**Authors:** Li Zhao, Chuanfu Yao, Hui Wang, Ji Dong, Jing Zhang, Xinping Xu, Haoyu Wang, Binwei Yao, Ke Ren, Liu Sun, Ruiyun Peng

**Affiliations:** Beijing Institute of Radiation Medicine, Beijing 100850, China; lillyliz@163.com (L.Z.); yaomax2010@126.com (C.Y.); wanghui597bj@163.com (H.W.); djtjwj@163.com (J.D.); zhang115614@163.com (J.Z.); xxpbjhd@163.com (X.X.); smart106@126.com (H.W.); ybwcsq@163.com (B.Y.); okayke1@163.com (K.R.); 13103249606@163.com (L.S.)

**Keywords:** microwave, radiation, immune response, transcriptomic, proteomic

## Abstract

With the rapidly increasing application of microwave technologies, the anxiety and speculation about microwave induced potential health hazards has been attracting more and more attention. In our daily life, people are exposed to complex environments with multi-frequency microwaves, especially L band and C band microwaves, which are commonly used in communications. In this study, we exposed rats to 1.5 GHz (L10), 4.3 GHz (C10) or multi-frequency (LC10) microwaves at an average power density of 10 mW/cm^2^. Both single and multi-frequency microwaves induced slight pathological changes in the thymus and spleen. Additionally, the white blood cells (WBCs) and lymphocytes in peripheral blood were decreased at 6 h and 7 d after exposure, suggesting immune suppressive responses were induced. Among lymphocytes, the B lymphocytes were increased while the T lymphocytes were decreased at 7 d after exposure in the C10 and LC10 groups, but not in the L10 group. Moreover, multi-frequency microwaves regulated the B and T lymphocytes more strongly than the C band microwave. The results of transcriptomics and proteomics showed that both single and multi-frequency microwaves regulated numerous genes associated with immune regulation and cellular metabolism in peripheral blood and in the spleen. However, multi-frequency microwaves altered the expression of many more genes and proteins. Moreover, multi-frequency microwaves down-regulated T lymphocytes’ development, differentiation and activation-associated genes, while they up-regulated B lymphocytes’ activation-related genes. In conclusion, multi-frequency microwaves of 1.5 GHz and 4.3 GHz produced immune suppressive responses via regulating immune regulation and cellular metabolism-associated genes. Our findings provide meaningful information for exploring potential mechanisms underlying multi-frequency induced immune suppression.

## 1. Introduction

Microwave technology, non-ionizing electromagnetic radiation ranging from 300 MHz to 300 GHz, has been widely used in various fields, such as mobile communication, medicine, industrial synthesis and so on [1,2,3,4]. In addition, the anxiety and speculation about the potential health hazards caused by microwaves have been growing in recent years [5,6]. In the past decades, most of the researchers aimed to uncover the biological effects caused by microwaves with a single frequency at indicated power density [7,8]. Our group has previously reported that S band microwaves, ranging from 2 GHz to 4 GHz, could cause significant injuries on several organs and tissues, including the nervous system, cardiovascular system, reproductive system and immune system [9,10,11,12]. Moreover, the potential underlying mechanisms were explored [7,13,14]. However, people are always exposed to complex microwave environment with different frequencies or power densities in daily life [7,8]. For example, the remote sensing satellites constantly equipped with synthetic aperture radar simultaneously emit a signal at frequencies of L-band (1–2 GHz), C-band (4–8 GHz) and X-band (8–12 GHz) [1]. Therefore, it is important to explore the biological effects and underlying mechanisms caused by multi-frequency microwaves.

The immune system is the first line of defense against pathogens and foreign molecules such as bacteria, viruses and mutated or dead cells. It has been widely demonstrated that the immune system is one of the most sensitive tissues to both ionizing and non-ionizing radiation [15,16]. Unlike ionizing radiation, the biological effects of non-radiation on immune cells, tissues and organs are controversial. Limited data suggested that microwaves could affect immune functions, including phagocytosis, proliferation of immune cells and antibody production [17,18]. However, opposite results might be presented when exposed to microwaves with different parameters, such as average power density, exposure period and so on [19,20].

Non-ionizing radiation, including microwave radiation, could produce both thermal effects and non-thermal effects. We have previously established an animal model exposed to low frequency and low power density to evaluate the non-thermal effects. In this study, we exposed Wistar rats to multi-frequency microwaves of 1.5 GHz (L band) and 4.3 GHz (C band) at an average power density of 10 mW/cm^2^. We showed that multi-frequency microwaves slightly injured the thymus and spleen, but not the bone marrow, which resulted in the decrease in white blood cells (WBC) and lymphocytes. Multi-frequency microwave induced immune suppressions were also demonstrated by transcriptomic and proteomic analysis. Further studies should be conducted to clarify the underlying mechanisms according to the results of transcriptomics and proteomics.

## 2. Results

### 2.1. Multi-Frequency Microwaves Caused Histopathological Injuries to Immune System

**Figure 1:**
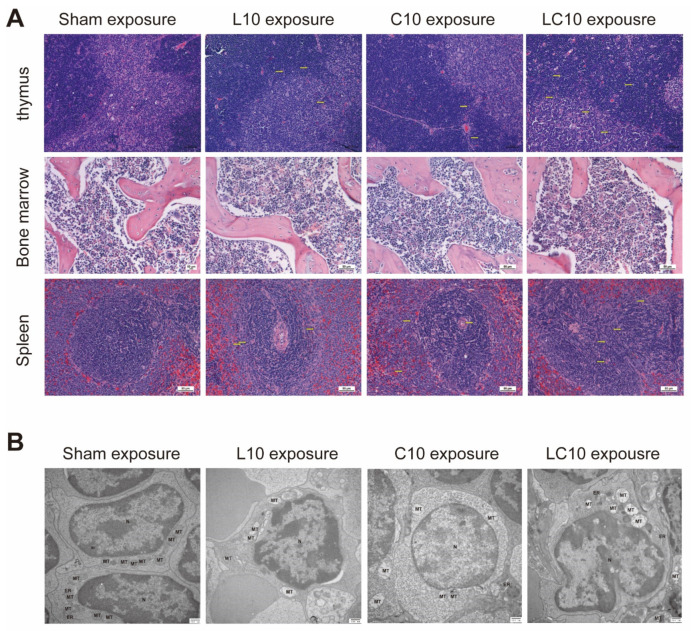
Microwave induced histopathological changes in thymus, bone marrow and spleen. 6–8-week male Wistar rats were exposed to microwaves with L band, C band and compound bands at the average power density of 10 mW/cm^2^. (**A**) At 6 h, 7 d, 14 d and 28 d after exposure, the rats were euthanized and the corresponding tissues were removed. The structure of the thymus, bone marrow and spleen was analyzed by hematoxylin–eosin (H&E) staining. The representative images from thymus, bone marrow and spleen at 7 d after microwave exposure were presented. The morphological changes were marked with yellow arrows. (**B**) For analyzing the ultrastructure of the spleen, the rats were euthanized and the spleens were removed at 7 d after exposure. The tissues were fixed in 2.5% glutaraldehyde and 1% osmium acid in sequence, the tissue blocks were processed with graded ethyl alcohols and embedded in EPON618. 70 nm thin slices were laid on copper mesh and then stained with heavy metals, uranyl acetate and lead citrate. The ultra-structures of the spleen were analyzed by transmission electron microscopy (TEM). N: nuclear; MT: mitochondria; ER: endoplasmic reticulum.

The immune system is composed of the central immune system (CIS) and peripheral immune system (PIS). In this study, the histopathological changes to the thymus, bone marrow and spleen, from both CIS and PIS, were analyzed by hematoxylin–eosin (H&E) staining. Significant pathological changes, such as congestion, could be detected in both the thymus and spleen at 6 h and 7 d after exposure, and were then gradually restored to normal at 14 d and 28 d after exposure. Furthermore, multi-frequency microwaves caused much more impressive injuries than other microwave exposed groups. However, no obvious injuries were observed in bone marrow at the indicated time points after exposure (Figure 1A).

Furthermore, we also evaluated the ultra-structural injuries in the spleen by transmission electron microscopy (TEM) at 7 d after exposure. In the Sham group, the chromatin was well-arranged in the nucleus, the endoplasmic reticulum was well-distributed in size and shape and the cristae of the mitochondria were transversely and longitudinally arranged. Microwave exposure resulted in swollen mitochondria and cavitation, especially in multi-frequency microwave exposed rats (Figure 1B). Microwave exposure-induced histopathological changes were closely associated with functional injuries of immune cells.

### 2.2. Multi-Frequency Microwave Induced Immune Suppression in Peripheral Blood

**Figure 2:**
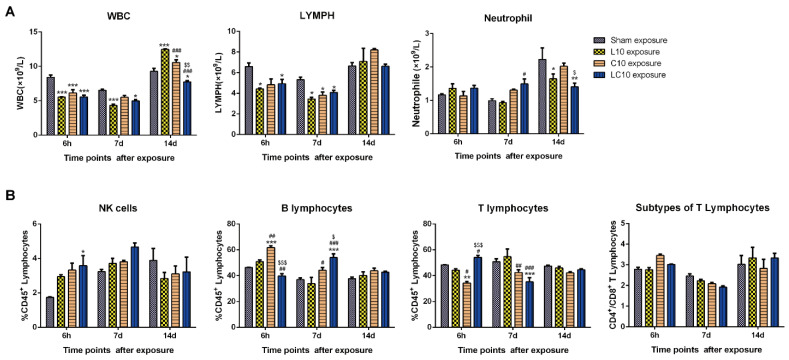
Microwave dynamically regulated immune cells in peripheral blood of rats. At 6 h, 7 d and 14 d after exposure to 10 mW/cm^2^ microwaves, the rats in the sham (Sham) group, L band (L10) group, C band (C10) group and compound bands (LC10) microwave exposed groups were anaesthetized by pentobarbital sodium and the peripheral blood was collected from inferior vena cava (IVC). The immune cells in peripheral blood, including white blood cells (WBC), neutrophils, and lymphocytes (LYMPH), were counted by automatic blood cell counter (**A**). Moreover, the percentage of natural killers (NK) cells, B lymphocytes, T lymphocytes among LYMPH, as well as the ratio of CD4^+^ T lymphocytes to CD8^+^ T lymphocytes, were analyzed by flow cytometry (**B**). Data were shown as mean ± s.e.m. *, *p* < 0.05, **, *p* < 0.01, ***, *p* < 0.001 vs. Sham group; #, *p* < 0.05, ##, *p* < 0.01, ###, *p* < 0.001 vs. L10 group; $, *p* < 0.05, $$, *p* < 0.01, $$$, *p* < 0.001 vs. C10 group.

Generally, 10 mW/cm^2^ microwaves reduced both the number of white blood cells (WBC) and lymphocytes at 6 h and 7 d after radiation, while WBC was decreased much more in L10 and LC10 groups (*p* < 0.05). However, the WBC in L10 (*p* < 0.001) and C10 (*p* < 0.05) groups was obviously increased at 14 d after exposure, which might be attributed to the compensatory mechanism of hematopoiesis. Interestingly, the WBC in the LC10 group was still lower than that in the Sham group (*p* < 0.05), suggesting that multi-frequency microwaves caused more severe injuries. Moreover, only multi-frequency microwaves up-regulated neutrophil at 7 d after exposure (*p* < 0.05), indicating that multi-frequency microwaves might induce inflammatory responses. The mechanisms underlying down-regulation of neutrophils at 14 d in the L10 (*p* < 0.05) and LC10 (*p* < 0.01) groups should be further investigated (Figure 2A).

Lymphocytes are the main effector cells in the immune system, and they are classified into several subtypes according to their development, surface markers and immune functions. Therefore, we analyzed three major subtypes of lymphocytes, including natural killers (NK), B lymphocytes and T lymphocytes. NK cells are pivotal effector cells of the innate immune system, and they can rapidly respond to viral infection and malignancy, as well as other stimuli. We showed that microwaves increased the percentage of NK cells at 6 h and 7 d after radiation, and significant up-regulation could only be detected in the LC10 group at 6 h after exposure (*p* < 0.05). Both B lymphocytes and T lymphocytes are the critical components of the adaptive immune system. In this study, we analyzed the percentage of B and T lymphocytes among CD45^+^ lymphocytes after microwave exposure. We found that C band, but not L band, microwaves up-regulated B lymphocytes (*p* < 0.001) and reduced the percentage of T lymphocytes (*p* < 0.01) at 6 h after exposure, which might be attributed to stress responses. Importantly, both C band and multi-frequency microwaves increased B lymphocytes and decreased T lymphocytes at 7 d after exposure. Additionally, impressive changes could be observed in the LC10 group (*p* < 0.001). However, both B and T lymphocytes were restored to normal levels at 14 d after exposure due to the activation of recovery mechanisms. T lymphocytes could be divided into CD4^+^ T and CD8^+^ T lymphocytes, which could exert immune functions mainly through cytokines release and direct cytotoxicity, respectively. The ratio of CD4^+^ and CD8^+^ T lymphocytes was not affected by both single and multi-frequency microwave exposure (Figure 2B). Our results suggested that both C band and multi-frequency microwaves could alter the balance of the immune system.

### 2.3. Multi-Frequency Microwave Transiently Increased Cytokines in Peripheral Blood

**Figure 3:**
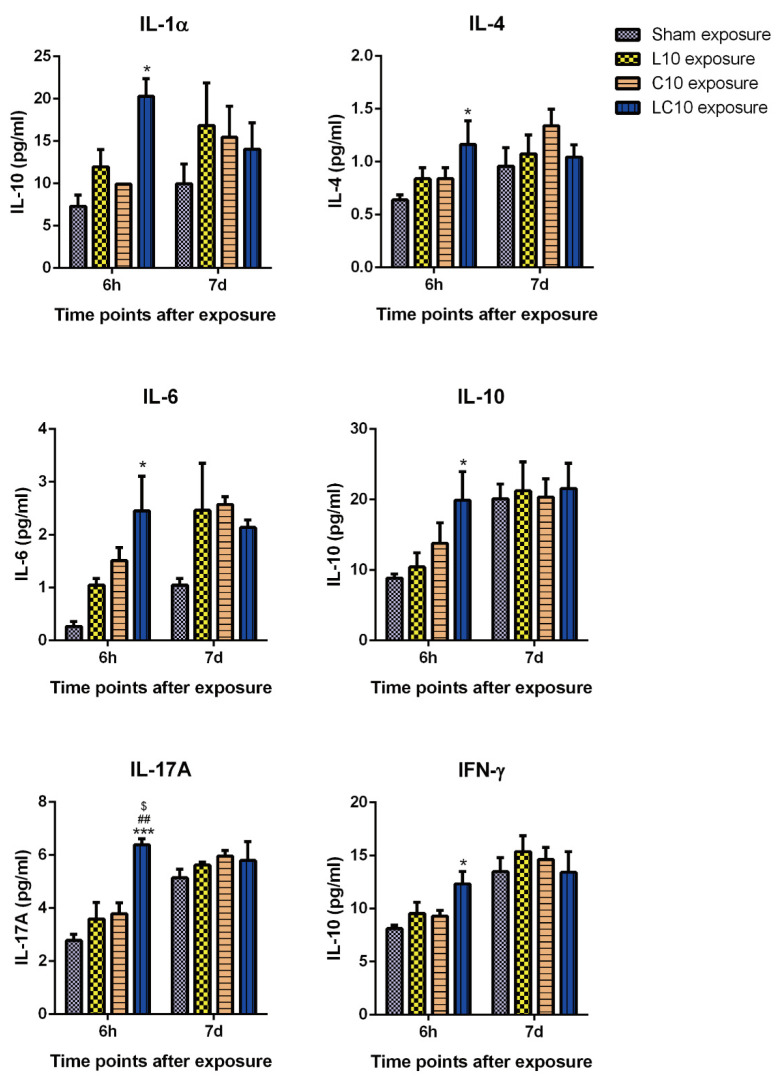
Compound microwaves increased cytokines expression in peripheral blood. At 6 h and 7 d after microwave exposure, the rats in the sham (Sham) group, L band (L10) group, C band (C10) group and compound bands (LC10) group were anaesthetized and the peripheral blood was collected from IVC. The serum was separated from peripheral blood by centrifugation. Then, the level of various cytokines, including interleukin (IL)-1α, IL-4, IL-6, IL-10, IL-17A and interferon γ (IFN-γ), was detected by flow cytometry using Cytokine&Chemokine 22-Plex Rat ProcartaPlex^TM^ Panel. Data were shown as mean ± s.e.m. *, *p* < 0.05, ***, *p* < 0.001 vs. Sham group; ##, *p* < 0.01 vs. L10 group; $, *p* < 0.05 vs. C10 group.

Immune cells respond to viral infections, external stimuli and cell mutations via various mechanisms, such as direct cytotoxicity, induction of cellular apoptosis, as well as release of cytokines and chemokines. Here, we analyzed the level of cytokines in peripheral blood by flow cytometry. No obvious alterations could be detected in peripheral blood flow, both at 6 h and 7 d after radiation with single frequency microwaves, including L10 and C10. However, multi-frequency microwaves could transiently increase the expression of interleukin (IL)-1α (*p* < 0.05), IL-4 (*p* < 0.05), IL-6 (*p* < 0.05), IL-10 (*p* < 0.05), IL-17A (*p* < 0.001) and interferon γ (IFN-γ) (*p* < 0.05) at 6 h after radiation. Then, these cytokines were rapidly restored to normal levels at 7 d after exposure (Figure 3). Our data suggested that multi-frequency microwaves could only transiently and slightly regulate the cytokines’ expression, which might be a complementary response to the decrease in lymphocytes after radiation.

### 2.4. Multi-Frequency Microwave Modulated Numerous Immune Associated Genes on mRNA Level

**Figure 4:**
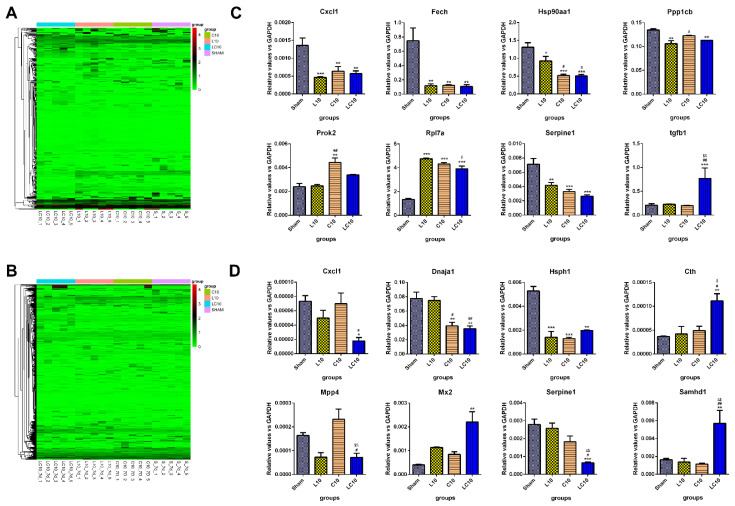
Transcriptomic analysis of differentially expressed genes (DEGs) in both peripheral blood and the spleen at 7 d after microwave exposure. At 7 d after microwave exposure, the peripheral blood and the spleen were collected from Sham group, L10 group, C10 group and LC10 group, as described in Section 4. The cDNA library was constructed, and the quality of cDNA library was confirmed by using the Agilent high sensitivity DNA assay on a Bioanalyzer 2100 system. Then, high throughput sequencing was conducted to investigate DEGs. The DEGs in peripheral blood (**A**) and the spleen (**C**) were analyzed by DESeq2 and were presented, respectively. Moreover, eight representative DEGs from peripheral blood (including Cxcl1, Fech, Hsp90aa1, Ppp1cb, Prok2, Rpl7a, Serpine1 and tgfb1) (**B**) and the spleen (including Cxcl1, Dnaja1, Hsph1, Cth, Mpp4, Mx2, Serpine1 and Samhd1) (**D**) were selected and then verified by real-time reverse transcription polymerase chain reaction (RT-PCR). Data were shown as mean ± s.e.m. *, *p* < 0.05, **, *p* < 0.01, ***, *p* < 0.001 vs. Sham group; #, *p* < 0.05, ##, *p* < 0.01 vs. L10 group; $, *p* < 0.05, $$, *p* < 0.01 vs. C10 group.

To investigate the potential mechanisms underlying microwave-induced immune suppression, transcriptomics was conducted to analyze the differential expressed genes (DEGs) in immune cells from both peripheral blood and the spleen. Generally, multi-frequency microwaves caused more DEGs both in peripheral blood and the spleen than single frequency microwaves. Additionally, the DEGs in the spleen (n = 1122) were much more than that in peripheral blood (n = 185) (Figure 4A,C, Table 1, Supplementary Figure S1). Eight DEGs were selected and verified by real-time reverse transcription polymerase chain reaction (RT-PCR) in both peripheral blood and the spleen (Figure 4B,D). Our results were consistent with the transcriptomics, especially in the LC10 group.

DEGs from peripheral blood exposed to L band, C band and multi-frequency microwaves were involved in 32, 46 and 425 categoric Gene Ontology (GO) items and participated in 1, 21 and 41 signaling Kyoto Encyclopedia of Genes and Genomes (KEGG) pathways, respectively (Table 2). Importantly, a number of DEGs were closely related with the immune processes and regulations, such as T cell differentiation, activation and proliferation, B cell maturation, activation and proliferation, cytokines’ release and so on. For example, up-regulated DEGs in the LC10 group participated in B cell affinity maturation and the B cell receptor signaling pathway and were also involved in negative regulation of T cell proliferation. These results were consistent with the alteration of B and T lymphocytes in peripheral blood after microwave exposure. Moreover, the down-regulated genes participated in Th17 cell differentiation and the IL-17 signaling pathway, suggesting that the functions of T lymphocytes were inhibited by multi-frequency microwaves (Supplementary Table S1).

Gene set enrichment analysis showed that DEGs in the spleen from L10, C10 and LC10 groups were included in 92, 312 and 382 categoric GO items and participated in 11, 29 and 43 signaling KEGG pathways, respectively (Table 3). We showed that down-regulated DEGs were involved in positive regulation of cytotoxic T cell differentiation, T-helper cell differentiation and immunoglobulin production, while up-regulated DEGs participated in negative regulation of macrophage activation. Moreover, KEGG analysis showed that the down-regulated DEGs also participated in Th17 cell differentiation, IL-17 signaling pathway and antigen processing and presentation (Supplementary Table S2). Our data suggested the immune suppressive state in the spleen after microwave exposure.

### 2.5. Multi-Frequency Microwave Modulated Numerous Immune Associated Proteins

**Figure 5:**
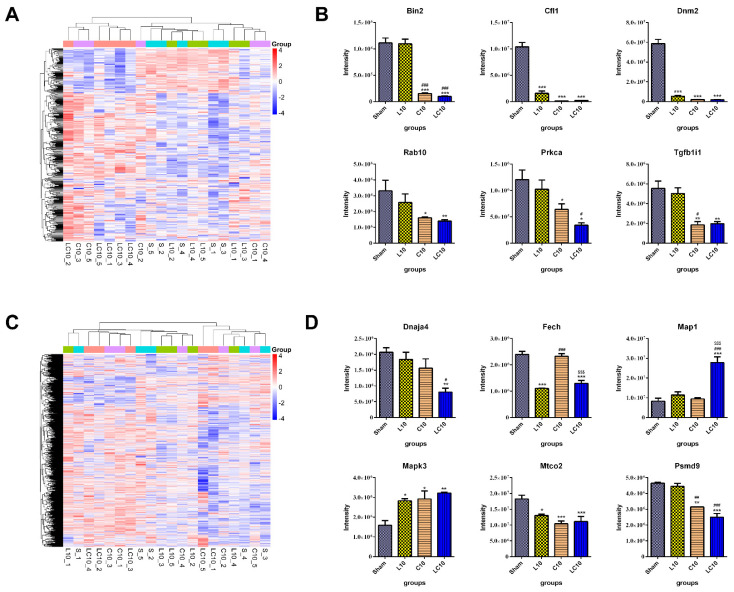
Proteomic analysis of differentially expressed proteins (DEPs) in peripheral blood and the spleen at 7 d after compound exposure. At 7 d after microwave exposure, the peripheral blood and the spleen were collected from the Sham group, L10 group, C10 group and LC10 group, as described in Section 4. The proteins were caught by proteominer beads from the sera of peripheral blood and spleen lysates, respectively. After enzymatic degradation and desalination, a spectral library was constructed by using a high pH reversed−phase fractionator in data−dependent acquisition (DDA) mode. Then, the samples were measured by data independent acquisition (DIA). The DEPs in peripheral blood (**A**) and the spleen (**C**) were presented, respectively. Six DEPs, both in peripheral blood (including Bin2, Cfl1, Dnm2, Rab10, Prkca and Tgfb1i1) (**B**) and in the spleen (including Dnaja4, Fech, Map1, Mapk3, Mtco2 and Psmd9) (**D**), were selected for verification by parallel reaction monitoring (PRM). Data were shown as mean ± s.e.m. *, *p* < 0.05, **, *p* < 0.01, ***, *p* < 0.001 vs. Sham group; #, *p* < 0.05, ##, *p* < 0.01, ###, *p* < 0.001 vs. L10 group; $$$, *p* < 0.001 vs. C10 group.

Proteomic analysis showed that 18, 86 and 78 up-regulated proteins and 28, 140 and 155 down-regulated proteins were detected in the peripheral blood after exposure to L band, C band and multi-frequency microwaves, respectively. Contrary to transcriptomic analysis, the differentially expressed proteins (DEPs) in the spleen were less than those in peripheral blood, which might be attributed to the complexity of peripheral blood (Figure 5A,C, Table 4, Supplementary Figure S2, Supplementary Files S1 and S2). Selected DEPs from peripheral blood and the spleen were verified by mass spectrum (Figure 5B,D).

The DEPs in peripheral blood were classified into 170, 177 and 150 categoric GO items and were involved in 10, 12 and 25 KEGG pathways after being exposed to L band, C band and multi-frequency microwaves, respectively (Table 5). These DEPs were closely associated with protein metabolism and transport, including proteasomal protein catabolic process, proteasomal ubiquitin-independent protein catabolic process, proteasome-mediated ubiquitin-dependent protein catabolic process, positive regulation of protein binding, endoplasmic reticulum to Golgi vesicle-mediated transport and intracellular protein transport. KEGG analysis suggested that DEPs participated in the signaling pathways, including regulation of actin cytoskeleton, proteasome, cGMP-PKG signaling pathway, RNA transport, NOD-like receptor signaling pathway and IL-17 signaling pathway (Supplementary Table S3).

Distinguishing from those in peripheral blood, DEPs in the spleen were closely related with the immune processes, which were involved in 384, 256 and 313 categoric GO items and 39, 15 and 9 KEGG pathways, respectively (Table 6). Down-regulated DEPs in the spleen were involved in immune associated biological processes, including T cell differentiation in the thymus, NK cell differentiation, response to IL-2, cellular response to IL-4, cellular response to virus and cellular response to DNA damage stimulus. Moreover, up-regulated DEPs participated in immune related biological processes, such as negative regulation of T cell proliferation, positive regulation of B cell activation and the B cell receptor signaling pathway. Moreover, KEGG analysis also showed that several immune and metabolism associated proteins were regulated by microwaves, including the complement and coagulation cascades, pyrimidine metabolism, pentose phosphate pathway, mRNA surveillance pathway and so on (Supplementary Table S4). Besides direct injuries on immune associated molecules, the changes in cellular metabolism also might be a potential mechanism underlying microwave induced immune dysfunctions.

## 3. Discussion

In recent years, the potential health hazards caused by microwaves have been attracting more and more attention. The World Health Organization (WHO) had classified electromagnetic field radiation as a new form of environmental pollution and “possibly carcinogenic to humans” [2,3]. Microwave exposure could induce biological responses in various tissues via both thermal and non-thermal mechanisms [4]. Our group has been focusing on microwave-induced non-thermal effects. We previously reported that microwaves could reduce learning and memory ability and decrease the synaptic plasticity of the hippocampus, as well as impair cardiac function in animal models [5,6,7,8]. Now, we are greatly interested in investigating biological responses to multi-frequency microwaves [5,9,10].

The immune system could rapidly respond to external and internal stimuli and play pivotal roles in protecting the body against viral infection, as well as cancer initiation and development. It has been reported that hematopoietic cells are more sensitive to microwaves than cells in many other tissues [11]. Microwave ablation has been used as a potential adjuvant therapy for cancer therapy. Clinical studies have suggested that microwave ablation induced anti-tumor effects, both through directly destroying tumor tissues and via activating immune responses in patients. Moreover, thermal effects have been recognized as the major mechanism for immune activation during microwave ablation [12,13,14,15]. However, potential non-thermal effects of microwaves on immune cells were also investigated in animal models after exposure to low-level electromagnetic field [16,17]. Our previous in vitro study demonstrated that microwaves induced cellular apoptosis and impaired cytotoxicity in NK cells [18]. In this study, we found that both single and multi-frequency microwaves decreased WBC and lymphocytes in peripheral blood, indicating that immune suppressions were induced by microwaves.

With the growing application of microwave technologies in our daily life, people are constantly under long-term environmental exposure to multi-frequency microwave radiation. For example, the satellite communication system and synthetic aperture radar simultaneously emit multi-frequency microwaves located in the L and C bands [1,19,20]. Therefore, we evaluated the biological effects on the immune system in rats after exposure to multi-frequency microwaves of 1.5 GHz (L band) and 4.3 GHz (C band) with the average power density of 10 mW/cm^2^. Our data suggested that both single and multi-frequency microwave exposure evoked immune suppressive responses, such as down-regulation of WBC and lymphocytes in peripheral blood. Otherwise, no obvious difference could be detected between single and multi-frequency exposure. However, we found that multi-frequency microwaves could decrease the percentage of T lymphocytes and increase the percentage of B lymphocytes much more strongly than single frequency microwaves, suggesting that T lymphocytes might be more sensitive to multi-frequency microwaves.

The cytokines and chemokines secreted from other tissues could stimulate or inhibit immune cells, while the immune cells in peripheral blood could enter into various tissues. The complex interactions between the immune system and other tissues increase the difficulties in studying the immune regulatory mechanisms. To clarify the mechanisms underlying microwave mediated immune dysfunctions, researchers evaluated the genotoxicity, cell proliferation and level of calcium in immune cells in vitro, as well as immunologically relevant parameters in vivo. However, controversial results have been reported due to differences in exposure parameters, immune cells and evaluation methods [17,21,22,23,24,25,26]. Both transcriptomics and proteomics are efficient and high throughput tools for basic studies and have been widely used in investigating potential mechanisms. In this study, we showed that multi-frequency microwaves induced much more DEGs and DEPs than single frequency microwaves in the spleen, suggesting that multi-frequency microwaves produced much stronger biological effects on the spleen. Similar results were also observed in DEGs from peripheral blood at the transcriptional level. Unexpectedly, the number of DEPs in peripheral blood after single or multi-frequency microwave radiation were at the similar level. Moreover, the DEPs in peripheral blood were much greater than that in the spleen, which was contrary to the DEGs. We speculated that the DEPs in peripheral blood were not only derived from the expression of immune cells, but were also secreted from other tissues. In addition, proteins secreted from other tissues could in turn stimulate or inhibit protein expression in immune cells. Therefore, the DEPs in peripheral blood might be a potential indicator for the whole body, not only for the immune system. Furthermore, bioinformatics analysis suggested that several T lymphocytes’ development, differentiation and activation associated genes were down-regulated both in peripheral blood and in the spleen, while B lymphocytes’ activation related genes were up-regulated, indicating that T lymphocytes were much more sensitive to multi-frequency microwaves. However, the mechanisms should be further investigated.

In conclusion, both 1.5 GHz (L band) and 4.3 GHz (C band) microwaves could cause immune suppression at the average power density of 10 mW/cm^2^. However, multi-frequency microwaves of 1.5 GHz and 4.3 GHz produced much more impressive immune suppressive responses, especially in inhibiting T lymphocytes. The transcriptomic and proteomic analysis suggested that multi-frequency microwaves regulated numerous genes associated with immune activation and metabolism, both at the mRNA and protein level. Moreover, several T lymphocytes’ development, differentiation and activation associated genes were down-regulated, while B lymphocytes’ activation related genes were up-regulated. Our data provided useful information for exploring potential mechanisms underlying multi-frequency induced immune suppression.

## 4. Materials and Methods

### 4.1. Animals

One hundred six- to eight-week-old male Wistar rats (180 g ± 20 g) (Vital River Laboratory Animal Technology, Beijing, China) were randomly divided into four groups: sham-exposed group (Sham), 1.5 GHz microwave (L10) exposed group, 4.3 GHz microwave (C10) exposed group and multi-frequency (1.5 GHz and 4.3 GHz) microwave (LC10) exposed group. The animal experiments were approved by the Institutional Animal Care and Use Committee at Beijing Institute of Radiation Medicine (Beijing, China), and all experimental procedures were performed in accordance with the National Institutes of Health Guide for the Care and Use of Laboratory Animals.

### 4.2. Microwave Exposure

The microwave exposure system, based on a klystron amplifier model JD 2000 (Vacuum Electronics Research Institute, Beijing, China), had been previously reported [5,8]. Rats in L10, C10 and LC10 groups were exposed to microwaves of 1.5 GHz, 4.3 GHz and multi-frequency (1.5 GHz and 4.3 GHz), respectively, at the average power intensity of 10 mW/cm^2^. Briefly, rats in microwave exposed groups were fixed in fan-shaped boxes, which were made of plexiglass and free of metal, and were exposed in the far field of a 12 dB-gain horn antenna. The power density was measured with a calibrated waveguide antenna over an area of 10 cm intervals horizontally on the plane with respect to the rat’s position. The distribution of the microwave density was described previously [5]. Rats in the Sham group were processed in parallel with those in the microwave exposed groups, but without microwave exposure.

### 4.3. Phenotypes of Immune Cells

At 6 h, 7 d and 14 d after microwave exposure, the rats in Sham, L10, C10 and LC10 groups were anaesthetized by 1% pentobarbital sodium at 30 mg/kg. Peripheral blood was collected from IVC. The immune cells in peripheral blood, including WBC, lymphocytes and neutrophils, were counted by automatic blood cell counter (Sysmex XN-1000, Kobe City, Japan).

Moreover, the subtypes of lymphocytes were detected by flow cytometry (C6, BD, Franklin Lake, NJ, USA) after being labeled with the corresponding antibody panels, as followsd: Panel 1 for NK cells (CD45, CD3, CD161A), Panel 2 for T lymphocytes and B lymphocytes (CD45, CD3, CD45R), Panel 3 for the phenotype of T lymphocytes (CD4, CD8a) (eBioscience, San Diego, CA, USA).

### 4.4. Secretion of Cytokines

At 6 h and 7 d after microwave exposure, peripheral blood was collected as described above. The sera were separated by centrifugation and the expression of interleukin (IL)-1α, IL-4, IL-6, IL-10, IL-17A and IFN-γ was analyzed by Cytokine&Chemokine 22-Plex Rat ProcartaPlex^TM^ Panel (Invitrogen, Carlsbad, CA, USA).

### 4.5. Histopathological Analysis

At 6 h, 7 d, 14 d and 28 d after microwave exposure, rats in each group were euthanized and the corresponding tissues were removed. For the thymus and the spleen, tissues were fixed in 10% buffered formalin solution, embedded in paraffin and cut at 3 μm thick in the coronal plane. For bone marrow, tissues were fixed with Helly solution immediately after isolation for 24 h, and thereafter fixed with 10% buffered formalin solution for 3 weeks, followed by decalcification for another 3 weeks. Then, the tissues were embedded in paraffin and cut at 3 μm thick in the coronal plane. The sections were stained with H&E, and the histopathological changes were observed by light microscopy (Leica, Wetzler, Germany).

### 4.6. Transmission Electron Microscopy (TEM)

The spleens of rats were collected at 7 d after microwave exposure, as described above. After being fixed in 2.5% glutaraldehyde and 1% osmium acid in sequence, the tissue blocks were processed with graded ethyl alcohols and embedded in EPON618. Seventy nm thin slices were laid on copper mesh and were then stained with heavy metals, uranyl acetate and lead citrate. The ultra-structures of the spleen in each group were observed and photographed by TEM (Hitachi, Tokyo, Japan).

### 4.7. Transcriptomics Analysis

The peripheral blood and spleen were collected at 7 d after exposure. Total RNA was isolated using the Trizol Reagent (Invitrogen Life Technologies, Carlsbad, CA, USA), and cDNA library was constructed by using NEBNext^®^ Ultra™ Directional RNA Library Prep Kit for Illumina^®^ (NEB, Ipswich, MA, USA). Then, the cDNA library was purified (AMPure XP system, Beckman Coulter, Brea, FL, USA) and quantified using the Agilent high sensitivity DNA assay on a Bioanalyzer 2100 system (Agilent, Palo Alto, CA, USA). Finally, the samples were then sequenced on a NovaSeq 6000 platform (Illumina, San Diego, CA, USA). The DEGs were analyzed by DESeq2 with screened conditions as follows: expression difference multiple |log_2_FoldChange| > 1, significant *q*-value ≤ 0.05.

Then, we used topGO to perform GO enrichment analysis to find the GO term with significantly enriched DEGs (*p* < 0.05), the *p*-value of which was calculated by hypergeometric distribution method. The enrichment analysis of KEGG pathway of DEGs also focused on the significant enrichment pathway with *p* < 0.05.

### 4.8. Proteomics Analysis

The peripheral blood and spleen were collected at 7 d after exposure. The proteins were extracted, and protein concentration was determined using the Bradford method. A 100 μg extracted protein from each sample was mixed, enzymatic degraded and desalinated. For spectral library generation, the proteomics analyses were performed using an U3000 UHPLC system (Thermo Fisher, Waltham, MA, USA) coupled with an Orbitrap fusion mass spectrometer (Thermo Fisher, Waltham, MA, USA) operating in the DDA mode. While the proteomics analysis of each sample was operated in DIA, the acquisition method consisted of one MS1 scan (350 to 1350 *m*/*z*, resolution 120,000 maximum injection time 50 ms, AGC target 4E5) and 60 segments at varying isolation windows from 349 *m*/*z* to 1500 *m*/*z*. Stepped normalized collision energy was 35. Data analysis was carried out as described in Bruder et al., with minor modifications [27]. Briefly, data extraction and extraction window were set to “dynamic” with correction factor 1 and identification was set to “normal distribution *p*-value estimator” with *q*-value cutoff of 0.01. The profiling strategy was set to “iRT profiling” with *q*-value cutoff of 0.01. Ultimately, protein inference was set to “from search engine”, protein quantity was set to “Average precursor quantity” and smallest quantitative unit was set to “Precursor ion” (summed fragment ions).

GO and InterPro (IPR) analysis were conducted using the interproscan-5 program against the non-redundant protein database and the databases Clusters of Orthologous Groups (COG). The KEGG was used to analyze the protein family and pathway. The enrichment pipeline was used to perform the enrichment analysis of GO and KEGG.

### 4.9. Real-Time Reverse Transcription Polymerase Chain Reaction (RT-PCR)

The peripheral blood and spleen were collected at 7 d after exposure. The total RNA was isolated by the Trizol Reagent (Invitrogen Life Technologies, Carlsbad, CA, USA) from peripheral blood and the spleen, and then the cDNA was synthesized by using revert aid first strand cDNA synthesis kit (Thermo Fisher Scientific, Waltham, MA, USA). The expression of indicated DEGs was analyzed by real-time RT-PCR using 2 × RealStar Green Fast Mixture with ROX II (Genstar, Beijing, China) on 7500 Fast Real-Time PCR System (Applied Biosystem Technologies, Foster City, CA, USA). The relative expression of DEGs was calculated by 2^−∆CT^, using rat glyceraldehyde-3-phosphate dehydrogenase (GAPDH) as control. The primers for each gene were shown in Supplementary Table S5.

### 4.10. Mass Spectrometry Analysis

Protein samples for mass spectrometry analysis were prepared as described above. After protein extraction, digestion and desalination, the samples were purified by U3000 UHPLC system (Thermo Fisher Scientific, Waltham, MA, USA). The expressions of DEPs were detected by parallel reaction monitoring (PRM).

### 4.11. Statistical Analysis

Data were presented as mean ± s.e.m. and analyzed by GraphPad Prism software version 6.0 (GraphPad software, San Diego, CA, USA). Grouped data were analyzed by one-way ANOVA followed by Bonferroni post hoc tests. Longitudinal data were analyzed by a two-way repeated measure ANOVA followed by Bonferroni post hoc tests. Differences were considered significant at two-sided *p* < 0.05.

## Figures and Tables

**Table 1:** The number of differentially expressed genes (DEGs) after compound microwave exposure in peripheral blood and the spleen.

Groups	Peripheral Blood		Spleen	
	Up-Regulated DEGs	Down-Regulated DEGs	Up-Regulated DEGs	Down-Regulated DEGs
L10 vs. Sham	44	41	381	453
C10 vs. Sham	47	47	423	521
LC10 vs. Sham	109	76	523	599
C10 vs. L10	31	42	449	425
LC10 vs. L10	58	63	552	482
LC10 vs. C10	70	60	393	435

**Table 2:** The gene set enrichment analysis of DEGs after compound microwave exposure in peripheral blood.

Groups	The Number of Categoric Items from GO Analysis		The Number of Categoric Signaling Pathways from KEGG Analysis	
	Up-Regulated DEGs	Down-Regulated DEGs	Up-Regulated DEGs	Down-Regulated DEGs
L10 vs. Sham	32	0	1	0
C10 vs. Sham	2	44	1	20
LC10 vs. Sham	277	148	27	14
C10 vs. L10	0	6	0	0
LC10 vs. L10	97	32	3	7
LC10 vs. C10	311	139	10	14

**Table 3:** The gene set enrichment analysis of DEGs after compound microwave exposure in the spleen.

Groups	The Number of Categoric Items from GO Analysis		The Number of Categoric Signaling Pathways from KEGG Analysis	
	Up-Regulated DEGs	Down-Regulated DEGs	Up-Regulated DEGs	Down-Regulated DEGs
L10 vs. Sham	45	47	6	5
C10 vs. Sham	91	221	9	20
LC10 vs. Sham	132	250	28	15
C10 vs. L10	38	44	3	3
LC10 vs. L10	181	104	25	4
LC10 vs. C10	61	21	3	22

**Table 4:** The number of differentially expressed proteins (DEPs) after compound microwave exposure in peripheral blood and the spleen.

Groups	Peripheral Blood		Spleen	
	Up-Regulated DEPs	Down-Regulated DEPs	Up-Regulated DEPs	Down-Regulated DEPs
L10 vs. Sham	18	28	31	17
C10 vs. Sham	86	140	27	24
LC10 vs. Sham	78	155	47	106
C10 vs. L10	59	22	18	29
LC10 vs. L10	39	100	40	84
LC10 vs. C10	12	11	27	35

**Table 5:** The gene set enrichment analysis of DEPs after compound microwave exposure in peripheral blood.

Groups	The Number of Categoric Items from GO Analysis		The Number of Categoric Signaling Pathways from KEGG Analysis	
	Up-Regulated DEPs	Down-Regulated DEPs	Up-Regulated DEPs	Down-Regulated DEPs
L10 vs. Sham	44	126	5	5
C10 e vs. Sham	26	151	3	9
LC10 vs. Sham	39	111	5	20
C10 vs. L10	51	77	1	4
LC10 vs. L10	44	129	4	10
LC10 vs. C10	28	91	2	7

**Table 6:** The gene set enrichment analysis of DEPs after compound microwave exposure in the spleen.

Groups	The Number of Categoric Items from GO Analysis		The Number of Categoric Signaling Pathways from KEGG Analysis	
	Up-Regulated DEPs	Down-Regulated DEPs	Up-Regulated DEPs	Down-Regulated DEPs
L10 vs. Sham	207	177	24	15
C10 vs. Sham	179	77	8	7
LC10 vs. Sham	149	164	4	5
C10 vs. L10	108	133	3	0
LC10 vs. L10	132	190	4	8
LC10 vs. C10	112	136	5	3

## Data Availability

The mass spectrometry proteomics data have been deposited to the ProteomeXchange Consortium (http://proteomecentral.proteomexchange.org (accessed on 1 May 2022)) via the iProX partner repository with the dataset identifier PXD034706.

## Supplementary Materials

The following supporting information can be downloaded at: https://www.mdpi.com/article/10.3390/ijms23136949/s1.
