# Supplementary material for: Immune Responses to Multi-Frequencies of 1.5 GHz and 4.3 GHz Microwave Exposure in Rats: Transcriptomic and Proteomic Analysis
Source: Int J Mol Sci. 2022 Jun 22;23(13):6949. doi: 10.3390/ijms23136949 (PMC9266614; doi:10.3390/ijms23136949)
Supplement: Supplementary file 1 [file ijms-23-06949-s001.zip › Supplementary Figure.docx]

**Supplementary Figure**

**
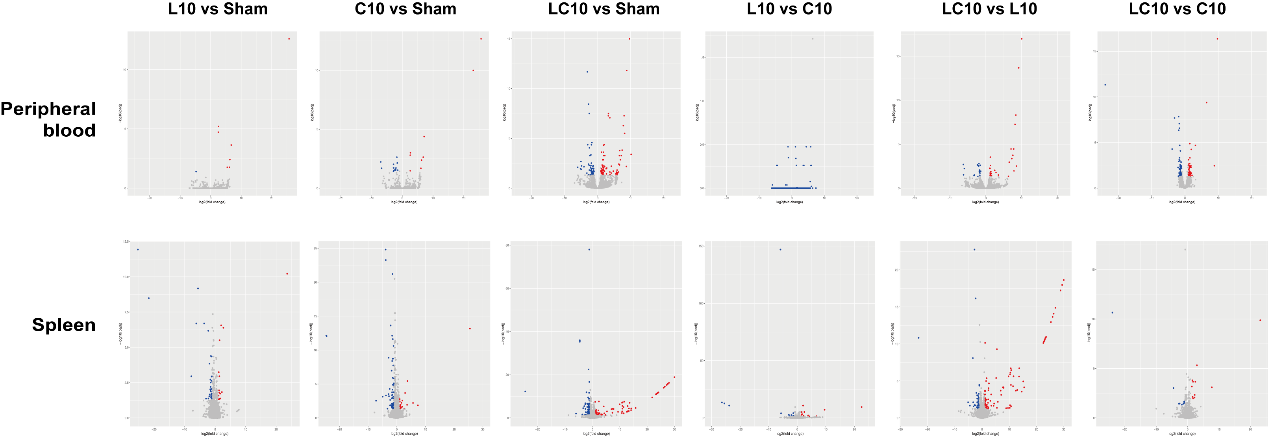
**

**Supplementary Figure S1 The volcano plots of transcriptomic analysis both in peripheral blood and spleen after multi-frequency microwave exposure.** The up-regulated genes (Red) and down-regulated genes (Blue) among corresponding groups were shown.

**
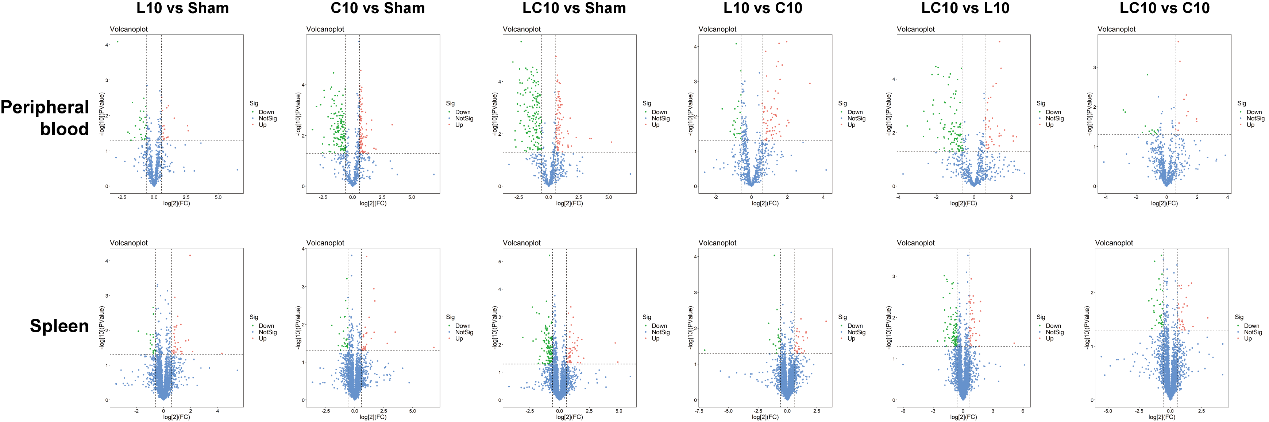
**

**Supplementary Figure S2 The volcano plots of proteomics analysis both in peripheral blood and spleen after multi-frequency microwave exposure.** The up-regulated proteins and down-regulated proteins were shown.
