# Supplementary material for: Immune Responses to Multi-Frequencies of 1.5 GHz and 4.3 GHz Microwave Exposure in Rats: Transcriptomic and Proteomic Analysis
Source: Int J Mol Sci. 2022 Jun 22;23(13):6949. doi: 10.3390/ijms23136949 (PMC9266614; doi:10.3390/ijms23136949)
Supplement: Supplementary file 1 [file ijms-23-06949-s001.zip › Supplementary File S3.docx]

**1. Proteomics analysis**

***Total Protein Extraction***

Samples were minced individually with liquid nitrogen and lysed in lysis buffer containing 50 mM NH_4_HCO_3_ (pH 7.4), 10 mM MgCl_2_, 7 M urea, 2 M thiourea, followed by 5 min of ultrasonication on ice. The lysate was centrifuged at 12000 g for 15 min at 4°C and the supernatant was transferred to a clean tube. Protein concentration was determined by Bradford protein assay.

***Peptide Preparation***

100 μ g protein from each sample were reduced with 10 mM DTT for 1 h at 56°C, and subsequently alkylated with sufficient Iodoacetamide for 1 h at room temperature in the dark. The protein was digested with Trypsin Gold (Promega) at 1:50 enzyme-to-substrate ratio. After 16 h of digestion at 37°C, part of peptides from samples were mixed equally. The mixture sample (mix-sample) and the remaining peptides (single-sample) were all desalted with C18 cartridge to remove the high urea, and desalted samples were dried by vacuum centrifugation.

***HPLC Fractionation***

The mix-sample was fractionated using a C18 column (Waters BEH C18 4.6×250 mm, 5 μm) on a Rigol L3000 HPLC operating at 1 mL/min, the column oven was set as 50°C. Mobile phases A (2% acetonitrile, adjusted pH to 10.0 using ammonium hydroxide) and B (98% acetonitrile, adjusted pH to 10.0 using ammonium hydroxide) were used to develop a gradient elution. The solvent gradient was set as follows: 3%B, 5 min; 3-8% B, 0.1 min; 8-18% B, 11.9 min; 18-32% B, 11 min; 32-45% B, 7 min; 45-80% B, 3 min; 80% B, 5 min; 80-5%, 0.1 min, 5% B, 6.9 min. The eluates were monitored at UV 214 nm, collected for a tube per minute and merged into 6 fractions finally. All fractions were dried under vacuum and reconstituted in 0.1% (v/v) formic acid (FA) in water. Add 0.2 μL standard peptides to the fraction sample for subsequent analyses.

***Library Construction-DDA mode***

For transition library construction, shotgun proteomics analyses were performed using an U3000 UHPLC system (Thermo Fisher) coupled with an Orbitrap fusion mass spectrometer (Thermo Fisher) operating in the data-dependent acquisition (DDA) mode. A sample volume containing 1 μg of total peptides from the fraction sample reconstituted in 0.1% FA was injected onto a home-made C18 Nano-Trap column (2 cm×100 μm, 3 μm). Peptides were separated on analytical column (25 cm×75 μm, 100 A), using a 120 min linear gradient from 0 to 100% of eluent B (0.08%FA in 80% ACN, 20% water) in eluent A (0.1%FA in water) at a flow rate of 350 nL/min. The detailed solvent gradient listed as follows: 0-4% B, 8 min; 4-10% B, 3min; 10-25% B, 77 min; 25-50% B, 10 min; 50-99% B, 10 min; 99-0% B, 12min. The Orbitrap Fusion mass spectrometer was operated in the data-dependent acquisition (DDA) mode using Xcalibur 3.0 software and there was a single full-scan mass spectrum in the Orbitrap (250-1450 m/z, 120,000 resolution) followed by 3 seconds data-dependent MS/MS scans in an Ion Routing Multipole at 30% normalized collision energy (HCD).

***LC-MS/MS Analysis-DIA mode***

The single-sample was reconstituted in 0.1% FA, mixed with 0.2 μL standard peptides (iRT kit, Biognosys), and injected onto U3000 UHPLC system (Thermo Fisher) coupled with an Orbitrap fusion mass spectrometer (Thermo Fisher) operating in the data-independent acquisition (DIA) mode. The liquid conditions were the same as above. For DIA acquisition, MS1 resolution was set to 120000, and MS2 resolution was set to 30000. The m/z range covered from 350 to 1350 m/z and variable 60 cycles. Full scan AGC target was set to 4×10^6^, injection time to 50 ms. DIA settings were NCE 35%, target value 1×10^6^ and maximum injection time was set to auto to allow the mass spectectometer always operating in the parallel ion filling and detection mode.

***The identification and quantitation of protein***

Data analysis and visualization of DDA and DIA data were performed using Proteome Discoverer 2.4 (PD 2.4, thermo) platform, Biognosys Spectronaut version 13, and R statistical framework. DDA MS raw files were analyzed by PD software (version 2.4) and peak lists were searched against protein database. Cysteine carbamidomethylation was set as a fixed modification and N-terminal acetylation and methionine oxidation as variable modifications. The false discovery rate was set to 5% for proteins and peptides, respectively and was determined by searching a reverse database. The enzyme specificity was set to trypsin (enabling cleavage before proline), and a maximum of two missed cleavages were allowed in the database search. Peptide identification was performed with an allowed initial precursor mass deviation up to 10 ppm and an allowed fragment mass deviation of 10 ppm. MS1-based label free quantification (LFQ) was done using maxLFQ algorithm[1].

MS2-based-label free quantification was carried out by analyzing DIA raw data using Biognosys Spectronaut (version 13) software. Data analysis was carried out as described in Bruder et al.[2] with minor modifications. Briefly, data extraction and extraction window were set to “dynamic” with correction factor 1, identification was set to “normal distribution p-value estimator” with q-value cutoff of 0.01. The profiling strategy was set to “iRT profiling” with q-value cutoff of 0.01. Ultimately, protein inference was set to “from search engine”, protein quantity was set to “Average precursor quantity” and smallest quantitative unit was set to “Precursor ion” (summed fragment ions).

***The functional analysis of protein and DEP***

Gene Ontology (GO) and InterPro (IPR) analysis were conducted using the interproscan-5 program against the non-redundant protein database, and the databases COG (Clusters of Orthologous Groups) and KEGG (Kyoto Encyclopedia of Genes and Genomes) were used to analyze the protein family and pathway.

**2. DEPs verification by parallel reaction monitoring (PRM)**

***Protein Extraction***

Samples were minced individually with liquid nitrogen and lysed in lysis buffer containing 50 mM NH_4_HCO_3_ pH 7.4, 10 mM MgCl_2_, 7 M urea, 2 M thiourea, followed by 5 min of ultrasonication on ice. The lysate was centrifuged at 12000 g for 15 min at 4°C and the supernatant was transferred to a clean tube. Protein concentration was determined by Bradford protein assay.

***Trypsin Digestion***

50 μg protein from each sample were reduced with 10 mM DTT for 1 h at 56°C, and subsequently alkylated with sufficient Iodoacetamide for 1 h at room temperature in the dark. The protein was digested with Trypsin Gold (Promega) at 1:25 enzyme-to-substrate ratio. After 16 h of digestion at 37°C, part of peptides from samples were mixed equally. The mixture sample (mix-sample) and the remaining peptides (single-sample) were all desalted with C18 cartridge to remove the high urea, and desalted samples were dried by vacuum centrifugation.

***Library Construction-DDA mode***

For transition library construction, shotgun proteomics analyses were performed using an Q Exactive HF-X mass spectrometer (Thermo Fisher) operating in the data-dependent acquisition (DDA) mode. A sample volume containing 1 μg of total peptides from the fraction sample reconstituted in 0.1% FA was injected onto a home-made C18 Nano-Trap column (2 cm×100 μm, 3 μm). Peptides were separated on analytical column (25 cm×75 μm, 100 A), using an 80 min linear gradient from 0 to 100% of eluent B (100% acetonitrile, 0.1% formic acid) and eluent A (100% water, 0.1% formic acid) at a flow rate of 600 nL/min. The detailed solvent gradient listed as follows: 8% B, 0min; 8-12% B, 7 min; 12-30% B, 48 min; 30-40% B, 10min; 40-95% B, 15 min.

The Q Exactive HF-X mass spectrometer was operated in the data-dependent acquisition mode using Xcalibur3.0 software and there is a single full-scan mass spectrum in the Orbitrap (350-1500 m/z, 120,000 resolution) followed by data-dependent MS/MS scans in an Ion Routing Multipole at 27% normalized collision energy (HCD).

***LC-MS/MS Analysis-PRM mode***

The single-sample was reconstituted in 0.1% FA, and injected onto U3000 UHPLC system (Thermo Fisher) coupled with Q Exactive HF-X mass spectrometer (Thermo Fisher) operating in the PRM mode. The liquid conditions were the same as above. For PRM acquisition, MS1 resolution was set to 60000, and MS2 resolution was set to 60000. The m/z range covered from 150 to 2000 m/z. Full scan AGC target was set to 3×106, injection time to 200 ms. PRM settings were NCE 28%, target value 1×10^5^ and maximum injection time was 100 ms to allow the mass spectectometer always operating in the parallel ion filling and detection mode

***Data Analysis***

The resulting MS data were processed using Skyline (v.3.6). Peptide settings: enzyme was set as Trypsin [KR/P], Max missed cleavage set as 2. The peptide length was set as 8-25, Variable modification was set as Carbamidomethyl on Cys and oxidation on Met, and max variable modifications was set as 3. Transition settings: precursor charges were set as 2, 3, ion charges were set as 1, 2, ion types were set as b, y, p. The product ions were set as from ion 3 to last ion, the ion match tolerance was set as 0.02 Da.

1. Cox, J., et al., *Accurate proteome-wide label-free quantification by delayed normalization and maximal peptide ratio extraction, termed MaxLFQ.* Mol Cell Proteomics, 2014. **13**(9): p. 2513-26.

2. Bruderer, R., et al., *Extending the limits of quantitative proteome profiling with data-independent acquisition and application to acetaminophen-treated three-dimensional liver microtissues.* Mol Cell Proteomics, 2015. **14**(5): p. 1400-10.
