# Supplementary material for: Immune Responses to Multi-Frequencies of 1.5 GHz and 4.3 GHz Microwave Exposure in Rats: Transcriptomic and Proteomic Analysis
Source: Int J Mol Sci. 2022 Jun 22;23(13):6949. doi: 10.3390/ijms23136949 (PMC9266614; doi:10.3390/ijms23136949)
Supplement: Supplementary file 1 [file ijms-23-06949-s001.zip › Supplementary Table S1.docx]

**Supplementary Table S1 GO analysis of DEGs between LC10 exposure and Sham exposure in peripheral blood**

| **GO** | **ID** | **Categories** | **Number of DEGs** | **Name of genes** | **P value** |
| --- | --- | --- | --- | --- | --- |
| BP | GO:0019886 | antigen processing and presentation of exogenous peptide antigen via MHC class II | 4 | RT1-Ba,Unc93b1,Cd74,RT1-Bb | 2.41E-08 |
|  | GO:0016560 | protein import into peroxisome matrix, docking | 2 | -,Pex5 | 4.59E-05 |
|  | GO:0042130 | negative regulation of T cell proliferation | 3 | RT1-Ba,Tgfb1,RT1-Bb | 5.31E-05 |
|  | GO:0031396 | regulation of protein ubiquitination | 2 | -,- | 0.000192541 |
|  | GO:0045807 | positive regulation of endocytosis | 2 | Cd14,- | 0.000496938 |
|  | GO:0045582 | positive regulation of T cell differentiation | 2 | RT1-Ba,Cd74 | 0.000809447 |
|  | GO:0019646 | aerobic electron transport chain | 1 | COX3 | 0.0016758 |
|  | GO:0045002 | double-strand break repair via single-strand annealing | 1 | Rad52 | 0.001696345 |
|  | GO:1902949 | positive regulation of tau-protein kinase activity | 1 | - | 0.001704363 |
|  | GO:1903827 | regulation of cellular protein localization | 1 | - | 0.001704363 |
|  | GO:1905323 | telomerase holoenzyme complex assembly | 1 | - | 0.001704363 |
|  | GO:0090200 | positive regulation of release of cytochrome c from mitochondria | 2 | Bak1,Pla2g6 | 0.001929698 |
|  | GO:0071385 | cellular response to glucocorticoid stimulus | 2 | RT1-Ba,RT1-Bb | 0.002292614 |
|  | GO:0050853 | B cell receptor signaling pathway | 2 | Nfam1,- | 0.002697189 |
|  | GO:0048242 | epinephrine secretion | 1 | Ly6e | 0.002786197 |
|  | GO:0006508 | proteolysis | 3 | Cpa1,Napsa,- | 0.003106765 |
|  | GO:0002344 | B cell affinity maturation | 1 | RT1-Bb | 0.003215842 |
|  | GO:0009623 | response to parasitic fungus | 1 | RT1-Bb | 0.003215842 |
|  | GO:0046635 | positive regulation of alpha-beta T cell activation | 1 | RT1-Bb | 0.003215842 |
|  | GO:0002792 | negative regulation of peptide secretion | 1 | Cd74 | 0.003240512 |
|  | GO:0035691 | macrophage migration inhibitory factor signaling pathway | 1 | Cd74 | 0.003240512 |
|  | GO:0042117 | monocyte activation | 1 | Npy | 0.003400392 |
|  | GO:0032273 | positive regulation of protein polymerization | 1 | - | 0.003414187 |
|  | GO:0044346 | fibroblast apoptotic process | 1 | Bak1 | 0.003446941 |
|  | GO:0015990 | electron transport coupled proton transport | 1 | - | 0.00345645 |
|  | GO:0061365 | positive regulation of triglyceride lipase activity | 1 | Pnlip | 0.003768901 |
|  | GO:0002460 | adaptive immune response based on somatic recombination of immune receptors built from immunoglobulin superfamily domains | 1 | Tgfb1 | 0.003956624 |
|  | GO:0009817 | defense response to fungus, incompatible interaction | 1 | Tgfb1 | 0.003956624 |
|  | GO:0032667 | regulation of interleukin-23 production | 1 | Tgfb1 | 0.003956624 |
|  | GO:0032943 | mononuclear cell proliferation | 1 | Tgfb1 | 0.003956624 |
|  | GO:0060751 | branch elongation involved in mammary gland duct branching | 1 | Tgfb1 | 0.003956624 |
|  | GO:0061035 | regulation of cartilage development | 1 | Tgfb1 | 0.003956624 |
|  | GO:1901666 | positive regulation of NAD+ ADP-ribosyltransferase activity | 1 | Tgfb1 | 0.003956624 |
|  | GO:1901339 | regulation of store-operated calcium channel activity | 1 | Pla2g6 | 0.003958266 |
|  | GO:0030890 | positive regulation of B cell proliferation | 2 | Cd74,- | 0.004238052 |
|  | GO:0071549 | cellular response to dexamethasone stimulus | 2 | Tgfb1,RT1-Bb | 0.004814948 |
|  | GO:0007267 | cell-cell signaling | 2 | Npy,- | 0.004972503 |
|  | GO:1903364 | positive regulation of cellular protein catabolic process | 1 | - | 0.005081658 |
|  | GO:0008535 | respiratory chain complex IV assembly | 1 | COX3 | 0.005189625 |
|  | GO:0045585 | positive regulation of cytotoxic T cell differentiation | 1 | - | 0.005192861 |
|  | GO:0031468 | nuclear envelope reassembly | 1 | - | 0.005236908 |
|  | GO:0070050 | neuron cellular homeostasis | 1 | Chmp2b | 0.005270112 |
|  | GO:0090135 | actin filament branching | 1 | Coro1b | 0.00664637 |
|  | GO:0002352 | B cell negative selection | 1 | Bak1 | 0.006663418 |
|  | GO:0048597 | post-embryonic camera-type eye morphogenesis | 1 | Bak1 | 0.006663418 |
|  | GO:1900103 | positive regulation of endoplasmic reticulum unfolded protein response | 1 | Bak1 | 0.006663418 |
|  | GO:0002579 | positive regulation of antigen processing and presentation | 1 | RT1-Bb | 0.006666382 |
|  | GO:0016049 | cell growth | 2 | Tgfb1,Emp3 | 0.00671936 |
|  | GO:1902463 | protein localization to cell leading edge | 1 | Coro1b | 0.006767173 |
|  | GO:0045040 | protein insertion into mitochondrial outer membrane | 1 | - | 0.006912525 |
|  | GO:0006418 | tRNA aminoacylation for protein translation | 1 | - | 0.006933036 |
|  | GO:0007004 | telomere maintenance via telomerase | 1 | - | 0.006945293 |
|  | GO:2000343 | positive regulation of chemokine (C-X-C motif) ligand 2 production | 1 | Cd74 | 0.007177151 |
|  | GO:0045066 | regulatory T cell differentiation | 1 | Tgfb1 | 0.007530408 |
|  | GO:0060965 | negative regulation of gene silencing by miRNA | 1 | Tgfb1 | 0.007732598 |
|  | GO:2000394 | positive regulation of lamellipodium morphogenesis | 1 | Coro1b | 0.007869852 |
|  | GO:0046514 | ceramide catabolic process | 1 | Cel | 0.007896045 |
|  | GO:0002513 | tolerance induction to self antigen | 1 | Tgfb1 | 0.007896833 |
|  | GO:0019049 | evasion or tolerance of host defenses by virus | 1 | Tgfb1 | 0.007899498 |
|  | GO:1900126 | negative regulation of hyaluronan biosynthetic process | 1 | Tgfb1 | 0.007899498 |
|  | GO:0048298 | positive regulation of isotype switching to IgA isotypes | 1 | Tgfb1 | 0.007899498 |
|  | GO:0034316 | negative regulation of Arp2/3 complex-mediated actin nucleation | 1 | Coro1b | 0.008348994 |
|  | GO:0060315 | negative regulation of ryanodine-sensitive calcium-release channel activity | 1 | Clic2 | 0.008570101 |
|  | GO:0021955 | central nervous system neuron axonogenesis | 1 | - | 0.008691631 |
|  | GO:1902259 | regulation of delayed rectifier potassium channel activity | 1 | Kcnab1 | 0.008763421 |
|  | GO:0071315 | cellular response to morphine | 1 | RT1-Bb | 0.009419561 |
|  | GO:0032098 | regulation of appetite | 1 | Npy | 0.010075784 |
|  | GO:0042415 | norepinephrine metabolic process | 1 | Ly6e | 0.010309974 |
|  | GO:0010880 | regulation of release of sequestered calcium ion into cytosol by sarcoplasmic reticulum | 1 | Clic2 | 0.010310465 |
|  | GO:0039702 | viral budding via host ESCRT complex | 1 | Chmp2b | 0.010313863 |
|  | GO:0051099 | positive regulation of binding | 1 | Clic2 | 0.010328652 |
|  | GO:0051973 | positive regulation of telomerase activity | 1 | - | 0.010443222 |
|  | GO:0006903 | vesicle targeting | 1 | Ahi1 | 0.010444059 |
|  | GO:0019800 | peptide cross-linking via chondroitin 4-sulfate glycosaminoglycan | 1 | Bgn | 0.010485306 |
|  | GO:0002827 | positive regulation of T-helper 1 type immune response | 1 | RT1-Bb | 0.010557334 |
|  | GO:0008635 | activation of cysteine-type endopeptidase activity involved in apoptotic process by cytochrome c | 1 | Bak1 | 0.010595761 |
|  | GO:0002830 | positive regulation of type 2 immune response | 1 | Cd74 | 0.010688944 |
|  | GO:0002906 | negative regulation of mature B cell apoptotic process | 1 | Cd74 | 0.010735668 |
|  | GO:0002455 | humoral immune response mediated by circulating immunoglobulin | 1 | - | 0.011348507 |
|  | GO:0043932 | ossification involved in bone remodeling | 1 | Tgfb1 | 0.011390281 |
|  | GO:0042773 | ATP synthesis coupled electron transport | 1 | ND5 | 0.011476121 |
|  | GO:0009083 | branched-chain amino acid catabolic process | 1 | Bckdhb | 0.011615859 |
|  | GO:0031536 | positive regulation of exit from mitosis | 1 | Tgfb1 | 0.011625594 |
|  | GO:0030299 | intestinal cholesterol absorption | 1 | Pnlip | 0.011640766 |
|  | GO:0014832 | urinary bladder smooth muscle contraction | 1 | Pla2g6 | 0.011666777 |
|  | GO:0051152 | positive regulation of smooth muscle cell differentiation | 1 | Tgfb1 | 0.011740896 |
|  | GO:0030214 | hyaluronan catabolic process | 1 | Tgfb1 | 0.011742108 |
|  | GO:0050731 | positive regulation of peptidyl-tyrosine phosphorylation | 2 | Cd74,- | 0.01175826 |
|  | GO:0007182 | common-partner SMAD protein phosphorylation | 1 | Tgfb1 | 0.011772208 |
|  | GO:0006729 | tetrahydrobiopterin biosynthetic process | 1 | Spr | 0.01181428 |
|  | GO:2000304 | positive regulation of ceramide biosynthetic process | 1 | Pla2g6 | 0.011817136 |
|  | GO:0034154 | toll-like receptor 7 signaling pathway | 1 | Unc93b1 | 0.011828654 |
|  | GO:0030705 | cytoskeleton-dependent intracellular transport | 1 | Pgd; Kif1b | 0.01182866 |
|  | GO:0047497 | mitochondrion transport along microtubule | 1 | Pgd; Kif1b | 0.01182866 |
|  | GO:1902260 | negative regulation of delayed rectifier potassium channel activity | 1 | Kcnab1 | 0.011981483 |
|  | GO:0006809 | nitric oxide biosynthetic process | 1 | - | 0.012097697 |
|  | GO:0035845 | photoreceptor cell outer segment organization | 1 | Ahi1 | 0.012209928 |
|  | GO:0006414 | translational elongation | 1 | LOC100362751 | 0.013039272 |
|  | GO:0030031 | cell projection assembly | 1 | Capg | 0.013244995 |
|  | GO:1902188 | positive regulation of viral release from host cell | 1 | Chmp2b | 0.013801379 |
|  | GO:0060539 | diaphragm development | 1 | Kcnab1 | 0.013806872 |
|  | GO:0000187 | activation of MAPK activity | 2 | Cd74,- | 0.013907932 |
|  | GO:0010046 | response to mycotoxin | 1 | Bak1 | 0.013975746 |
|  | GO:0002606 | positive regulation of dendritic cell antigen processing and presentation | 1 | Cd74 | 0.014269188 |
|  | GO:0009620 | response to fungus | 1 | Bak1 | 0.014429793 |
|  | GO:0007183 | SMAD protein complex assembly | 1 | Tgfb1 | 0.014439055 |
|  | GO:0010248 | establishment or maintenance of transmembrane electrochemical gradient | 1 | Bak1 | 0.014454452 |
|  | GO:0070242 | thymocyte apoptotic process | 1 | Bak1 | 0.014512803 |
|  | GO:0035767 | endothelial cell chemotaxis | 1 | Coro1b | 0.014615753 |
|  | GO:0090238 | positive regulation of arachidonic acid secretion | 1 | Pla2g6 | 0.014798743 |
|  | GO:0001961 | positive regulation of cytokine-mediated signaling pathway | 1 | Cd74 | 0.015031497 |
|  | GO:0050861 | positive regulation of B cell receptor signaling pathway | 1 | Nfam1 | 0.015155755 |
|  | GO:0042482 | positive regulation of odontogenesis | 1 | Tgfb1 | 0.015188036 |
|  | GO:0051131 | chaperone-mediated protein complex assembly | 1 | - | 0.015390424 |
|  | GO:0007034 | vacuolar transport | 1 | Chmp2b | 0.01541263 |
|  | GO:0085029 | extracellular matrix assembly | 1 | Tgfb1 | 0.015478228 |
|  | GO:1901673 | regulation of mitotic spindle assembly | 1 | Chmp2b | 0.015513446 |
|  | GO:0016239 | positive regulation of macroautophagy | 1 | Scoc | 0.015563391 |
|  | GO:0034138 | toll-like receptor 3 signaling pathway | 1 | Unc93b1 | 0.015620554 |
|  | GO:0034162 | toll-like receptor 9 signaling pathway | 1 | Unc93b1 | 0.015620554 |
|  | GO:0033619 | membrane protein proteolysis | 1 | Napsa | 0.015649012 |
|  | GO:0006450 | regulation of translational fidelity | 1 | Rps5 | 0.015661314 |
|  | GO:0033365 | protein localization to organelle | 1 | Ahi1 | 0.015670689 |
|  | GO:0045667 | regulation of osteoblast differentiation | 1 | Hemgn | 0.015719718 |
|  | GO:0060364 | frontal suture morphogenesis | 1 | Tgfb1 | 0.015726937 |
|  | GO:0010936 | negative regulation of macrophage cytokine production | 1 | Tgfb1 | 0.015739283 |
|  | GO:0032471 | negative regulation of endoplasmic reticulum calcium ion concentration | 1 | Bak1 | 0.015765346 |
|  | GO:0043496 | regulation of protein homodimerization activity | 1 | Bak1 | 0.01695818 |
|  | GO:0002262 | myeloid cell homeostasis | 1 | Bak1 | 0.017005426 |
|  | GO:0006120 | mitochondrial electron transport, NADH to ubiquinone | 1 | - | 0.017007932 |
|  | GO:0045581 | negative regulation of T cell differentiation | 1 | Cd74 | 0.017214924 |
|  | GO:0007507 | heart development | 2 | Cacybp,Kcnab1 | 0.017305312 |
|  | GO:0060416 | response to growth hormone | 1 | Cacybp | 0.017310239 |
|  | GO:0048708 | astrocyte differentiation | 1 | Sox6 | 0.017313832 |
|  | GO:0010592 | positive regulation of lamellipodium assembly | 1 | - | 0.01732521 |
|  | GO:0016042 | lipid catabolic process | 2 | Pla2g6,Pnlip | 0.017823997 |
|  | GO:0031065 | positive regulation of histone deacetylation | 1 | Tgfb1 | 0.017829641 |
|  | GO:0002244 | hematopoietic progenitor cell differentiation | 2 | Tgfb1,Pld4 | 0.018094225 |
|  | GO:1902262 | apoptotic process involved in patterning of blood vessels | 1 | Bak1 | 0.018227112 |
|  | GO:0060744 | mammary gland branching involved in thelarche | 1 | Tgfb1 | 0.018629609 |
|  | GO:0001867 | complement activation, lectin pathway | 1 | Fcnb | 0.018733711 |
|  | GO:1902166 | negative regulation of intrinsic apoptotic signaling pathway in response to DNA damage by p53 class mediator | 1 | Cd74 | 0.018748199 |
|  | GO:0010225 | response to UV-C | 1 | Bak1 | 0.01877929 |
|  | GO:0051280 | negative regulation of release of sequestered calcium ion into cytosol | 1 | Tgfb1 | 0.018857813 |
|  | GO:0060762 | regulation of branching involved in mammary gland duct morphogenesis | 1 | Tgfb1 | 0.018869829 |
|  | GO:0060907 | positive regulation of macrophage cytokine production | 1 | Cd74 | 0.01893128 |
|  | GO:0001763 | morphogenesis of a branching structure | 1 | Tgfb1 | 0.018958959 |
|  | GO:0042176 | regulation of protein catabolic process | 1 | - | 0.018972386 |
|  | GO:0010524 | positive regulation of calcium ion transport into cytosol | 1 | Bak1 | 0.019067109 |
|  | GO:0032481 | positive regulation of type I interferon production | 1 | Cd14 | 0.019340762 |
|  | GO:0043497 | regulation of protein heterodimerization activity | 1 | Bak1 | 0.019658658 |
|  | GO:0035265 | organ growth | 1 | Ly6e | 0.020292192 |
|  | GO:0010824 | regulation of centrosome duplication | 1 | Chmp2b | 0.020573564 |
|  | GO:0010001 | glial cell differentiation | 1 | Metrn | 0.02069439 |
|  | GO:0045793 | positive regulation of cell size | 1 | - | 0.020797995 |
|  | GO:0006796 | phosphate-containing compound metabolic process | 1 | Tgfb1 | 0.021667872 |
|  | GO:0043129 | surfactant homeostasis | 1 | Napsa | 0.022159762 |
|  | GO:0042026 | protein refolding | 1 | - | 0.022217391 |
|  | GO:0000920 | cell separation after cytokinesis | 1 | Chmp2b | 0.022381315 |
|  | GO:0090037 | positive regulation of protein kinase C signaling | 1 | Pla2g6 | 0.022432646 |
|  | GO:0007270 | neuron-neuron synaptic transmission | 1 | Pgd; Kif1b | 0.022511362 |
|  | GO:0007435 | salivary gland morphogenesis | 1 | Tgfb1 | 0.023018029 |
|  | GO:0008354 | germ cell migration | 1 | Tgfb1 | 0.023034102 |
|  | GO:0050777 | negative regulation of immune response | 1 | Tgfb1 | 0.023159005 |
|  | GO:0045577 | regulation of B cell differentiation | 1 | Nfam1 | 0.023480794 |
|  | GO:0007184 | SMAD protein import into nucleus | 1 | Tgfb1 | 0.023510808 |
|  | GO:0050921 | positive regulation of chemotaxis | 1 | Tgfb1 | 0.023517538 |
|  | GO:0050765 | negative regulation of phagocytosis | 1 | Tgfb1 | 0.023541239 |
|  | GO:0070723 | response to cholesterol | 1 | Tgfb1 | 0.023731745 |
|  | GO:0045445 | myoblast differentiation | 1 | Kcnab1 | 0.024155081 |
|  | GO:0030010 | establishment of cell polarity | 1 | - | 0.024223813 |
|  | GO:0006754 | ATP biosynthetic process | 1 | Tgfb1 | 0.024241239 |
|  | GO:0050772 | positive regulation of axonogenesis | 1 | Metrn | 0.024295763 |
|  | GO:0050900 | leukocyte migration | 1 | - | 0.025082947 |
|  | GO:0031100 | animal organ regeneration | 2 | Bak1,Tgfb1 | 0.025224993 |
|  | GO:0032801 | receptor catabolic process | 1 | Tgfb1 | 0.025236862 |
|  | GO:0060179 | male mating behavior | 1 | Grn | 0.025389972 |
|  | GO:0043537 | negative regulation of blood vessel endothelial cell migration | 1 | Tgfb1 | 0.02552344 |
|  | GO:0048642 | negative regulation of skeletal muscle tissue development | 1 | Tgfb1 | 0.025541079 |
|  | GO:0031529 | ruffle organization | 1 | Coro1b | 0.025557354 |
|  | GO:0060314 | regulation of ryanodine-sensitive calcium-release channel activity | 1 | Jsrp1 | 0.025857786 |
|  | GO:0034616 | response to laminar fluid shear stress | 1 | Tgfb1 | 0.026011843 |
|  | GO:0031571 | mitotic G1 DNA damage checkpoint | 1 | Dgkz | 0.026023975 |
|  | GO:0032060 | bleb assembly | 1 | Emp3 | 0.026060896 |
|  | GO:0060068 | vagina development | 1 | Bak1 | 0.02611754 |
|  | GO:0035108 | limb morphogenesis | 1 | Bak1 | 0.026278289 |
|  | GO:0035988 | chondrocyte proliferation | 1 | Grn | 0.026297453 |
|  | GO:0060391 | positive regulation of SMAD protein import into nucleus | 1 | Tgfb1 | 0.026403798 |
|  | GO:0001783 | B cell apoptotic process | 1 | Bak1 | 0.026410239 |
|  | GO:0043518 | negative regulation of DNA damage response, signal transduction by p53 class mediator | 1 | Cd74 | 0.026424185 |
|  | GO:0007406 | negative regulation of neuroblast proliferation | 1 | Tgfb1 | 0.026633966 |
|  | GO:0006997 | nucleus organization | 1 | Chmp2b | 0.027067012 |
|  | GO:0006986 | response to unfolded protein | 1 | - | 0.027267865 |
|  | GO:0060389 | pathway-restricted SMAD protein phosphorylation | 1 | Tgfb1 | 0.027294829 |
|  | GO:0009060 | aerobic respiration | 1 | - | 0.027333432 |
|  | GO:0010629 | negative regulation of gene expression | 2 | Bak1,Tgfb1 | 0.027670645 |
|  | GO:0001934 | positive regulation of protein phosphorylation | 2 | Pla2g6,Tgfb1 | 0.02800577 |
|  | GO:0071223 | cellular response to lipoteichoic acid | 1 | Cd14 | 0.028353586 |
|  | GO:0032740 | positive regulation of interleukin-17 production | 1 | Tgfb1 | 0.028627871 |
|  | GO:0045059 | positive thymic T cell selection | 1 | Cd74 | 0.028669735 |
|  | GO:0016064 | immunoglobulin mediated immune response | 1 | Cd74 | 0.028949187 |
|  | GO:0017015 | regulation of transforming growth factor beta receptor signaling pathway | 1 | Tgfb1 | 0.029043296 |
|  | GO:0048675 | axon extension | 1 | - | 0.029440538 |
|  | GO:0045899 | positive regulation of RNA polymerase II transcriptional preinitiation complex assembly | 1 | Psmc4 | 0.029720045 |
|  | GO:0031334 | positive regulation of protein complex assembly | 1 | Tgfb1 | 0.030006395 |
|  | GO:0050832 | defense response to fungus | 1 | Cotl1 | 0.03004841 |
|  | GO:0032270 | positive regulation of cellular protein metabolic process | 1 | Tgfb1 | 0.030084046 |
|  | GO:1901800 | positive regulation of proteasomal protein catabolic process | 1 | Psmc4 | 0.03034671 |
|  | GO:0072657 | protein localization to membrane | 1 | Jsrp1 | 0.030353641 |
|  | GO:0061351 | neural precursor cell proliferation | 1 | Grn | 0.030471511 |
|  | GO:0001666 | response to hypoxia | 2 | ND5,- | 0.030526399 |
|  | GO:0051967 | negative regulation of synaptic transmission, glutamatergic | 1 | Pla2g6 | 0.030570329 |
|  | GO:0051085 | chaperone cofactor-dependent protein refolding | 1 | Cd74 | 0.031407248 |
|  | GO:0032930 | positive regulation of superoxide anion generation | 1 | Tgfb1 | 0.031966607 |
|  | GO:0001937 | negative regulation of endothelial cell proliferation | 1 | - | 0.032374212 |
|  | GO:0007568 | aging | 2 | Cacybp,- | 0.032391213 |
|  | GO:0055007 | cardiac muscle cell differentiation | 1 | Cacybp | 0.032485066 |
|  | GO:0010763 | positive regulation of fibroblast migration | 1 | Tgfb1 | 0.032528415 |
|  | GO:0050890 | cognition | 1 | Chmp2b | 0.032717665 |
|  | GO:2000679 | positive regulation of transcription regulatory region DNA binding | 1 | Tgfb1 | 0.03323987 |
|  | GO:0046902 | regulation of mitochondrial membrane permeability | 1 | Bak1 | 0.033630883 |
|  | GO:0042060 | wound healing | 2 | Tgfb1,Coro1b | 0.033688165 |
|  | GO:0000042 | protein targeting to Golgi | 1 | Gcc1 | 0.034328518 |
|  | GO:0015986 | ATP synthesis coupled proton transport | 1 | ATP8 | 0.034567125 |
|  | GO:2000249 | regulation of actin cytoskeleton reorganization | 1 | Tgfb1 | 0.035660332 |
|  | GO:0032700 | negative regulation of interleukin-17 production | 1 | Tgfb1 | 0.036188626 |
|  | GO:0042572 | retinol metabolic process | 1 | Cel | 0.036832495 |
|  | GO:0043968 | histone H2A acetylation | 1 | Brd8 | 0.036928287 |
|  | GO:0043536 | positive regulation of blood vessel endothelial cell migration | 1 | Tgfb1 | 0.037799878 |
|  | GO:0032026 | response to magnesium ion | 1 | Cd14 | 0.037966015 |
|  | GO:0035066 | positive regulation of histone acetylation | 1 | Tgfb1 | 0.038763474 |
|  | GO:0019835 | cytolysis | 1 | Bak1 | 0.03879394 |
|  | GO:0019370 | leukotriene biosynthetic process | 1 | Cotl1 | 0.03888374 |
|  | GO:0048535 | lymph node development | 1 | Tgfb1 | 0.039089578 |
|  | GO:0061025 | membrane fusion | 1 | - | 0.039276738 |
|  | GO:0008053 | mitochondrial fusion | 1 | Bak1 | 0.039306453 |
|  | GO:0050790 | regulation of catalytic activity | 1 | - | 0.039317315 |
|  | GO:0001974 | blood vessel remodeling | 1 | Bgn | 0.039418503 |
|  | GO:0034341 | response to interferon-gamma | 1 | RT1-Ba | 0.039537806 |
|  | GO:0006886 | intracellular protein transport | 3 | Tom1,Cd74,Rhog | 0.039725663 |
|  | GO:0045089 | positive regulation of innate immune response | 1 | Polr3b | 0.040040408 |
|  | GO:0070306 | lens fiber cell differentiation | 1 | Tgfb1 | 0.040773575 |
|  | GO:0051016 | barbed-end actin filament capping | 1 | Capg | 0.040839244 |
|  | GO:0045060 | negative thymic T cell selection | 1 | Cd74 | 0.040859432 |
|  | GO:0060999 | positive regulation of dendritic spine development | 1 | Grn | 0.04115178 |
|  | GO:0007274 | neuromuscular synaptic transmission | 1 | Pgd; Kif1b | 0.042162741 |
|  | GO:0002028 | regulation of sodium ion transport | 1 | Tgfb1 | 0.042268218 |
|  | GO:0022408 | negative regulation of cell-cell adhesion | 1 | Tgfb1 | 0.042346333 |
|  | GO:0007080 | mitotic metaphase plate congression | 1 | Chmp2b | 0.042828906 |
|  | GO:0010033 | response to organic substance | 2 | Tgfb1,- | 0.043358308 |
|  | GO:0008156 | negative regulation of DNA replication | 1 | Tgfb1 | 0.044126038 |
|  | GO:0055093 | response to hyperoxia | 1 | ATP8 | 0.044169201 |
|  | GO:0010800 | positive regulation of peptidyl-threonine phosphorylation | 1 | Tgfb1 | 0.044269001 |
|  | GO:0034605 | cellular response to heat | 1 | - | 0.04432549 |
|  | GO:0001516 | prostaglandin biosynthetic process | 1 | Cd74 | 0.04447666 |
|  | GO:0031000 | response to caffeine | 1 | - | 0.044567429 |
|  | GO:0007032 | endosome organization | 1 | Chmp2b | 0.044631006 |
|  | GO:0010811 | positive regulation of cell-substrate adhesion | 1 | Npy | 0.046126939 |
|  | GO:0042307 | positive regulation of protein import into nucleus | 1 | Tgfb1 | 0.046221026 |
|  | GO:0043011 | myeloid dendritic cell differentiation | 1 | Tgfb1 | 0.046293328 |
|  | GO:0071277 | cellular response to calcium ion | 1 | Cacybp | 0.046318782 |
|  | GO:0032728 | positive regulation of interferon-beta production | 1 | Polr3b | 0.046422876 |
|  | GO:0061436 | establishment of skin barrier | 1 | Cela2a | 0.046735753 |
|  | GO:0046677 | response to antibiotic | 1 | - | 0.047566188 |
|  | GO:0007492 | endoderm development | 1 | Tgfb1 | 0.047668659 |
|  | GO:0009409 | response to cold | 1 | - | 0.047705683 |
|  | GO:0050909 | sensory perception of taste | 1 | Npy | 0.048110112 |
|  | GO:0007631 | feeding behavior | 1 | Npy | 0.048678438 |
|  | GO:0001837 | epithelial to mesenchymal transition | 1 | Tgfb1 | 0.048691799 |
|  | GO:0006952 | defense response | 1 | Cd74 | 0.048799562 |
|  | GO:0048488 | synaptic vesicle endocytosis | 1 | Grn | 0.049305688 |
|  | GO:0045429 | positive regulation of nitric oxide biosynthetic process | 1 | - | 0.049493961 |
|  | GO:0045216 | cell-cell junction organization | 1 | Tgfb1 | 0.049700579 |
| CC | GO:0042613 | MHC class II protein complex | 3 | RT1-Ba,Cd74,RT1-Bb | 3.27E-06 |
|  | GO:0016020 | membrane | 11 | Bak1,Ifi47,-,Tom1,Tufm,Psmc4,Rps5,Cd79a,Rhog,Polr2b,Pex5 | 0.000375951 |
|  | GO:0005771 | multivesicular body | 2 | Cd74,RT1-Bb | 0.000999945 |
|  | GO:0005747 | mitochondrial respiratory chain complex I | 2 | ND5,- | 0.001664697 |
|  | GO:0097226 | sperm mitochondrial sheath | 1 | - | 0.001704363 |
|  | GO:0097524 | sperm plasma membrane | 1 | - | 0.001704363 |
|  | GO:0009897 | external side of plasma membrane | 4 | RT1-Ba,Cd74,RT1-Bb,- | 0.002006828 |
|  | GO:0035692 | macrophage migration inhibitory factor receptor complex | 1 | Cd74 | 0.003240512 |
|  | GO:0035693 | NOS2-CD74 complex | 1 | Cd74 | 0.003240512 |
|  | GO:0062023 | collagen-containing extracellular matrix | 1 | - | 0.003301508 |
|  | GO:1990635 | proximal dendrite | 1 | Kcnab1 | 0.003519098 |
|  | GO:0005746 | mitochondrial respiratory chain | 1 | - | 0.004966069 |
|  | GO:0043005 | neuron projection | 3 | Cacybp,ND5,- | 0.005161546 |
|  | GO:0044294 | dendritic growth cone | 1 | - | 0.005218091 |
|  | GO:0005778 | peroxisomal membrane | 2 | -,Pex5 | 0.00590411 |
|  | GO:0005829 | cytosol | 6 | Scoc,-,Chmp2b,Kcnab1,-,- | 0.006051351 |
|  | GO:0030877 | beta-catenin destruction complex | 1 | Cacybp | 0.006976349 |
|  | GO:0022627 | cytosolic small ribosomal subunit | 2 | Rps5,Rps16 | 0.007687749 |
|  | GO:0005947 | mitochondrial alpha-ketoglutarate dehydrogenase complex | 1 | Bckdhb | 0.007711219 |
|  | GO:0009986 | cell surface | 5 | Cd14,Tgfb1,Grn,Nfam1,RT1-Bb | 0.007776582 |
|  | GO:0045277 | respiratory chain complex IV | 1 | COX3 | 0.008477629 |
|  | GO:0042571 | immunoglobulin complex, circulating | 1 | - | 0.008840113 |
|  | GO:0017101 | aminoacyl-tRNA synthetase multienzyme complex | 1 | - | 0.010312622 |
|  | GO:0071004 | U2-type prespliceosome | 1 | Snrpb | 0.011331652 |
|  | GO:0032991 | protein-containing complex | 1 | - | 0.011801232 |
|  | GO:0000815 | ESCRT III complex | 1 | Chmp2b | 0.012047725 |
|  | GO:0034705 | potassium channel complex | 1 | Kcnab1 | 0.01220206 |
|  | GO:0005683 | U7 snRNP | 1 | Snrpb | 0.01282061 |
|  | GO:0005641 | nuclear envelope lumen | 1 | Cacybp | 0.013881908 |
|  | GO:0044224 | juxtaparanode region of axon | 1 | Kcnab1 | 0.01397072 |
|  | GO:0045121 | membrane raft | 3 | Cel,Cd14,Nfam1 | 0.013988866 |
|  | GO:0097208 | alveolar lamellar body | 1 | Napsa | 0.014588421 |
|  | GO:0071204 | histone pre-mRNA 3'end processing complex | 1 | Snrpb | 0.014842506 |
|  | GO:0019815 | B cell receptor complex | 1 | - | 0.01513886 |
|  | GO:0000276 | mitochondrial proton-transporting ATP synthase complex, coupling factor F(o) | 1 | ATP8 | 0.016315157 |
|  | GO:0036038 | TCTN-B9D complex | 1 | Ahi1 | 0.017422372 |
|  | GO:0005783 | endoplasmic reticulum | 5 | Bak1,-,Unc93b1,Cd74,Pld4 | 0.018304112 |
|  | GO:0046930 | pore complex | 1 | Bak1 | 0.01842045 |
|  | GO:0031595 | nuclear proteasome complex | 1 | Psmc4 | 0.018931737 |
|  | GO:0032839 | dendrite cytoplasm | 1 | Kcnab1 | 0.01903207 |
|  | GO:0090543 | Flemming body | 1 | Capg | 0.019323027 |
|  | GO:0046696 | lipopolysaccharide receptor complex | 1 | Cd14 | 0.019439526 |
|  | GO:0005753 | mitochondrial proton-transporting ATP synthase complex | 1 | ATP8 | 0.019540456 |
|  | GO:0009986 | cell surface | 3 | Bgn,-,- | 0.019978186 |
|  | GO:0044295 | axonal growth cone | 1 | - | 0.020882567 |
|  | GO:0005764 | lysosome | 3 | RT1-Ba,Cd74,Napsa | 0.020927177 |
|  | GO:0005686 | U2 snRNP | 1 | Snrpb | 0.021107999 |
|  | GO:0005777 | peroxisome | 2 | -,Pex5 | 0.022046035 |
|  | GO:0036126 | sperm flagellum | 1 | - | 0.022278756 |
|  | GO:0034709 | methylosome | 1 | Snrpb | 0.022323979 |
|  | GO:0000812 | Swr1 complex | 1 | Brd8 | 0.022411573 |
|  | GO:0008540 | proteasome regulatory particle, base subcomplex | 1 | Psmc4 | 0.022540678 |
|  | GO:0022624 | proteasome accessory complex | 1 | Psmc4 | 0.022581795 |
|  | GO:0005763 | mitochondrial small ribosomal subunit | 1 | Mrps18c | 0.02363568 |
|  | GO:0005687 | U4 snRNP | 1 | Snrpb | 0.025417228 |
|  | GO:0000015 | phosphopyruvate hydratase complex | 1 | Eno1 | 0.025535614 |
|  | GO:0031597 | cytosolic proteasome complex | 1 | Psmc4 | 0.026571743 |
|  | GO:0005682 | U5 snRNP | 1 | Snrpb | 0.030308267 |
|  | GO:0042588 | zymogen granule | 1 | Cel | 0.030430783 |
|  | GO:0034707 | chloride channel complex | 1 | Clic2 | 0.031182259 |
|  | GO:0005912 | adherens junction | 1 | Ahi1 | 0.032886912 |
|  | GO:0070062 | extracellular exosome | 11 | Snrpb,Tagln2,Capg,Tom1,Cotl1,Cd14,Tufm,Cd74,Rps5,Napsa,Coro1b | 0.033879205 |
|  | GO:0046540 | U4/U6 x U5 tri-snRNP complex | 1 | Snrpb | 0.034148598 |
|  | GO:0035267 | NuA4 histone acetyltransferase complex | 1 | Brd8 | 0.034533669 |
|  | GO:0005875 | microtubule associated complex | 1 | Pgd; Kif1b | 0.035247474 |
|  | GO:0034719 | SMN-Sm protein complex | 1 | Snrpb | 0.036604848 |
|  | GO:0005576 | extracellular region | 4 | Fcnb,Cela2a,Pnlip,Cpa2 | 0.036703047 |
|  | GO:0005665 | DNA-directed RNA polymerase II, core complex | 1 | Polr2b | 0.037310591 |
|  | GO:0030027 | lamellipodium | 2 | Dgkz,Coro1b | 0.037356773 |
|  | GO:0032588 | trans-Golgi network membrane | 1 | Pld4 | 0.040678066 |
|  | GO:0005685 | U1 snRNP | 1 | Snrpb | 0.042619983 |
|  | GO:0005852 | eukaryotic translation initiation factor 3 complex | 1 | Eif3k | 0.04286905 |
|  | GO:0031307 | integral component of mitochondrial outer membrane | 1 | Bak1 | 0.04314922 |
|  | GO:0016234 | inclusion body | 1 | Psmc4 | 0.043361383 |
|  | GO:0005801 | cis-Golgi network | 1 | - | 0.046271663 |
| MF | GO:0005052 | peroxisome matrix targeting signal-1 binding | 2 | -,Pex5 | 1.54E-05 |
|  | GO:0046982 | protein heterodimerization activity | 5 | RT1-a,Bak1,Eno1,Tgfb1,RT1-Bb | 5.16E-05 |
|  | GO:0004181 | metallocarboxypeptidase activity | 3 | Cpa1,Cpa2,Cpb1 | 5.76E-05 |
|  | GO:0032549 | ribonucleoside binding | 2 | Polr3b,Polr2b | 9.19E-05 |
|  | GO:0051020 | GTPase binding | 2 | -,- | 0.000157213 |
|  | GO:0004252 | serine-type endopeptidase activity | 5 | Prss3b,Cela2a,Ctrc,Cela3b,- | 0.000342263 |
|  | GO:0004806 | triglyceride lipase activity | 2 | Cel,Pnlip | 0.000516248 |
|  | GO:0004129 | cytochrome-c oxidase activity | 2 | COX3,- | 0.000838488 |
|  | GO:0044325 | ion channel binding | 2 | Kcnab1,- | 0.000858801 |
|  | GO:0008137 | NADH dehydrogenase (ubiquinone) activity | 2 | ND5,- | 0.000992028 |
|  | GO:0031841 | neuropeptide Y receptor binding | 1 | Npy | 0.001641929 |
|  | GO:0000150 | recombinase activity | 1 | Rad52 | 0.001696345 |
|  | GO:0070182 | DNA polymerase binding | 1 | - | 0.001704363 |
|  | GO:0003823 | antigen binding | 2 | Tgfb1,- | 0.002031485 |
|  | GO:0004757 | sepiapterin reductase activity | 1 | Spr | 0.003340202 |
|  | GO:1990782 | protein tyrosine kinase binding | 1 | - | 0.003357692 |
|  | GO:0097718 | disordered domain specific binding | 1 | - | 0.003392204 |
|  | GO:0002135 | CTP binding | 1 | - | 0.00346272 |
|  | GO:0030911 | TPR domain binding | 1 | - | 0.00346272 |
|  | GO:0008238 | exopeptidase activity | 1 | Cpa1 | 0.003854042 |
|  | GO:0050253 | retinyl-palmitate esterase activity | 1 | Cel | 0.003953158 |
|  | GO:0003899 | DNA-directed RNA polymerase activity | 2 | Polr3b,Polr2b | 0.004160627 |
|  | GO:0051022 | Rho GDP-dissociation inhibitor binding | 1 | - | 0.005196115 |
|  | GO:0017098 | sulfonylurea receptor binding | 1 | - | 0.005218091 |
|  | GO:0002134 | UTP binding | 1 | - | 0.005218091 |
|  | GO:0071208 | histone pre-mRNA DCP binding | 1 | Snrpb | 0.006513437 |
|  | GO:0030235 | nitric-oxide synthase regulator activity | 1 | - | 0.006864661 |
|  | GO:0004033 | aldo-keto reductase (NADP) activity | 1 | Kcnab1 | 0.006894471 |
|  | GO:0032564 | dATP binding | 1 | - | 0.006963753 |
|  | GO:0097367 | carbohydrate derivative binding | 1 | Fcnb | 0.007239343 |
|  | GO:0003826 | alpha-ketoacid dehydrogenase activity | 1 | Bckdhb | 0.007695559 |
|  | GO:0001727 | lipid kinase activity | 1 | Dgkz | 0.007901133 |
|  | GO:0048039 | ubiquinone binding | 1 | - | 0.008674144 |
|  | GO:0019955 | cytokine binding | 1 | Cd74 | 0.010210598 |
|  | GO:0048156 | tau protein binding | 1 | - | 0.010448933 |
|  | GO:0050998 | nitric-oxide synthase binding | 1 | Cd74 | 0.010823981 |
|  | GO:0042605 | peptide antigen binding | 2 | RT1-Ba,RT1-Bb | 0.011619817 |
|  | GO:0034713 | type I transforming growth factor beta receptor binding | 1 | Tgfb1 | 0.011647875 |
|  | GO:0004771 | sterol esterase activity | 1 | Cel | 0.011769961 |
|  | GO:0005114 | type II transforming growth factor beta receptor binding | 1 | Tgfb1 | 0.011827031 |
|  | GO:0034714 | type III transforming growth factor beta receptor binding | 1 | Tgfb1 | 0.011827031 |
|  | GO:0004518 | nuclease activity | 1 | Eme2 | 0.012201507 |
|  | GO:0008599 | protein phosphatase type 1 regulator activity | 1 | - | 0.013845458 |
|  | GO:0043008 | ATP-dependent protein binding | 1 | Pla2g6 | 0.014610078 |
|  | GO:0036402 | proteasome-activating ATPase activity | 1 | Psmc4 | 0.015037644 |
|  | GO:0070891 | lipoteichoic acid binding | 1 | Cd14 | 0.015155438 |
|  | GO:0047499 | calcium-independent phospholipase A2 activity | 1 | Pla2g6 | 0.015657029 |
|  | GO:0004983 | neuropeptide Y receptor activity | 1 | Npy | 0.01720562 |
|  | GO:0070402 | NADPH binding | 1 | Kcnab1 | 0.017311159 |
|  | GO:0097110 | scaffold protein binding | 1 | - | 0.017395081 |
|  | GO:0042803 | protein homodimerization activity | 3 | Bak1,Eno1,Tgfb1 | 0.018920172 |
|  | GO:0005184 | neuropeptide hormone activity | 1 | Npy | 0.019646702 |
|  | GO:0005539 | glycosaminoglycan binding | 1 | Bgn | 0.020902862 |
|  | GO:0004602 | glutathione peroxidase activity | 1 | Clic2 | 0.021799276 |
|  | GO:0043208 | glycosphingolipid binding | 1 | Cel | 0.022289041 |
|  | GO:0004622 | lysophospholipase activity | 1 | Cel | 0.023514777 |
|  | GO:0016538 | cyclin-dependent protein serine/threonine kinase regulator activity | 1 | Cks1b | 0.023615135 |
|  | GO:0004634 | phosphopyruvate hydratase activity | 1 | Eno1 | 0.025535614 |
|  | GO:0017025 | TBP-class protein binding | 1 | Psmc4 | 0.026691401 |
|  | GO:0019903 | protein phosphatase binding | 1 | - | 0.027499415 |
|  | GO:0051400 | BH domain binding | 1 | Bak1 | 0.028589815 |
|  | GO:0050840 | extracellular matrix binding | 1 | Bgn | 0.029509136 |
|  | GO:0042803 | protein homodimerization activity | 2 | -,- | 0.029520969 |
|  | GO:0005525 | GTP binding | 4 | Ifi47,Rabl6,Tufm,Rhog | 0.029884121 |
|  | GO:0015643 | toxic substance binding | 1 | RT1-Bb | 0.029930467 |
|  | GO:0043130 | ubiquitin binding | 1 | - | 0.03256981 |
|  | GO:0042826 | histone deacetylase binding | 1 | - | 0.032619627 |
|  | GO:0005244 | voltage-gated ion channel activity | 1 | Clic2 | 0.032868448 |
|  | GO:0051082 | unfolded protein binding | 1 | - | 0.033903686 |
|  | GO:0008574 | ATP-dependent microtubule motor activity, plus-end-directed | 1 | Pgd; Kif1b | 0.035053188 |
|  | GO:0001530 | lipopolysaccharide binding | 1 | Cd14 | 0.035527438 |
|  | GO:0015078 | hydrogen ion transmembrane transporter activity | 1 | ATP8 | 0.036408175 |
|  | GO:0004143 | diacylglycerol kinase activity | 1 | Dgkz | 0.038806015 |
|  | GO:0015459 | potassium channel regulator activity | 1 | Kcnab1 | 0.040644483 |
|  | GO:0005254 | chloride channel activity | 1 | Clic2 | 0.04142104 |
|  | GO:0000049 | tRNA binding | 1 | - | 0.046170825 |
|  | GO:0001664 | G protein-coupled receptor binding | 1 | Npy | 0.049442337 |
