# Supplementary material for: Immune Responses to Multi-Frequencies of 1.5 GHz and 4.3 GHz Microwave Exposure in Rats: Transcriptomic and Proteomic Analysis
Source: Int J Mol Sci. 2022 Jun 22;23(13):6949. doi: 10.3390/ijms23136949 (PMC9266614; doi:10.3390/ijms23136949)
Supplement: Supplementary file 1 [file ijms-23-06949-s001.zip › Supplementary Table S2.docx]

**Supplementary Table S2 GO analysis of DEGs between LC10 exposure and Sham exposure in spleen**

| **GO** | **ID** | **Categories** | **Number of DEGs** | **Name of genes** | **P value** |
| --- | --- | --- | --- | --- | --- |
| BP | GO:0007586 | digestion | 7 | Clps,LOC312273,Pnliprp2,-,-,-,Cckar | 1.63E-11 |
|  | GO:0042026 | protein refolding | 6 | Dnaja1,Dnaja4,Hspa1l,-,-,-, | 8.53E-11 |
|  | GO:1902949 | positive regulation of tau-protein kinase activity | 3 | -,-,- | 3.00E-08 |
|  | GO:1903364 | positive regulation of cellular protein catabolic process | 3 | -,-,- | 3.23E-07 |
|  | GO:1903827 | regulation of cellular protein localization | 3 | -,-,- | 3.83E-07 |
|  | GO:1905323 | telomerase holoenzyme complex assembly | 3 | -,-,- | 3.83E-07 |
|  | GO:0045585 | positive regulation of cytotoxic T cell differentiation | 3 | -,-,- | 4.50E-07 |
|  | GO:0032273 | positive regulation of protein polymerization | 3 | -,-,- | 4.98E-07 |
|  | GO:0045040 | protein insertion into mitochondrial outer membrane | 3 | -,-,- | 9.35E-07 |
|  | GO:0009408 | response to heat | 5 | Dnaja1,Dnaja4,-,-,- | 1.56E-06 |
|  | GO:0016042 | lipid catabolic process | 5 | Clps,Pnlip,Pnliprp1,Pnliprp2,Ins2 | 2.46E-06 |
|  | GO:0007004 | telomere maintenance via telomerase | 3 | -,-,- | 3.13E-06 |
|  | GO:0006809 | nitric oxide biosynthetic process | 3 | -,-,- | 4.69E-06 |
|  | GO:0021955 | central nervous system neuron axonogenesis | 3 | -,-,- | 4.86E-06 |
|  | GO:0051973 | positive regulation of telomerase activity | 3 | -,-,- | 6.78E-06 |
|  | GO:0010592 | positive regulation of lamellipodium assembly | 3 | -,-,- | 1.41E-05 |
|  | GO:0006508 | proteolysis | 5 | Cpa1,LOC312273,-,-,- | 1.69E-05 |
|  | GO:0051131 | chaperone-mediated protein complex assembly | 3 | -,-,- | 1.86E-05 |
|  | GO:0019064 | fusion of virus membrane with host plasma membrane | 4 | -,-,-,- | 2.55E-05 |
|  | GO:0019062 | virion attachment to host cell | 4 | -,-,-,- | 2.98E-05 |
|  | GO:0045793 | positive regulation of cell size | 3 | -,-,- | 3.99E-05 |
|  | GO:0031396 | regulation of protein ubiquitination | 3 | -,-,- | 4.20E-05 |
|  | GO:0030010 | establishment of cell polarity | 3 | -,-,- | 7.36E-05 |
|  | GO:0006986 | response to unfolded protein | 3 | -,-,- | 0.000121659 |
|  | GO:0048675 | axon extension | 3 | -,-,- | 0.000128236 |
|  | GO:0016052 | carbohydrate catabolic process | 2 | -,- | 0.000187316 |
|  | GO:0046677 | response to antibiotic | 3 | -,-,- | 0.000278419 |
|  | GO:0009409 | response to cold | 3 | -,-,- | 0.000337253 |
|  | GO:0045429 | positive regulation of nitric oxide biosynthetic process | 3 | -,-,- | 0.000345169 |
|  | GO:0050821 | protein stabilization | 4 | Bag3,-,-,- | 0.000348438 |
|  | GO:0034605 | cellular response to heat | 3 | -,-,- | 0.000372082 |
|  | GO:0001764 | neuron migration | 4 | Myh10,-,-,- | 0.000474514 |
|  | GO:0006457 | protein folding | 4 | Dnajb4,-,-,- | 0.000560939 |
|  | GO:0021670 | lateral ventricle development | 2 | Myh10,Tsku | 0.00066189 |
|  | GO:0090084 | negative regulation of inclusion body assembly | 2 | Dnaja4,Dnajb1 | 0.00069221 |
|  | GO:0051085 | chaperone cofactor-dependent protein refolding | 2 | Hsph1,Dnajb1 | 0.001114832 |
|  | GO:0033138 | positive regulation of peptidyl-serine phosphorylation | 3 | -,-,- | 0.00193339 |
|  | GO:0051897 | positive regulation of protein kinase B signaling | 3 | -,-,- | 0.002016791 |
|  | GO:0008625 | extrinsic apoptotic signaling pathway via death domain receptors | 2 | Bag3,Dedd2 | 0.002707259 |
|  | GO:0000281 | mitotic cytokinesis | 2 | Myh10,- | 0.002963381 |
|  | GO:0046034 | ATP metabolic process | 2 | Fignl1,Atp7a | 0.003167939 |
|  | GO:0046222 | aflatoxin metabolic process | 1 | Akr7a3 | 0.003385695 |
|  | GO:0046223 | aflatoxin catabolic process | 1 | Akr7a3 | 0.003385695 |
|  | GO:0070488 | neutrophil aggregation | 1 | S100a8 | 0.003410025 |
|  | GO:1904401 | cellular response to Thyroid stimulating hormone | 1 | - | 0.003412987 |
|  | GO:1904628 | cellular response to phorbol 13-acetate 12-myristate | 1 | - | 0.003412987 |
|  | GO:0044240 | multicellular organismal lipid catabolic process | 1 | Pla2g1b | 0.003510707 |
|  | GO:0031099 | regeneration | 1 | Reg1a | 0.003790003 |
|  | GO:0061365 | positive regulation of triglyceride lipase activity | 1 | Pnlip | 0.003815706 |
|  | GO:0038188 | cholecystokinin signaling pathway | 1 | Cckar | 0.003818983 |
|  | GO:0019068 | virion assembly | 3 | -,-,- | 0.003874722 |
|  | GO:0015819 | lysine transport | 1 | Slc7a3 | 0.003882049 |
|  | GO:0015822 | ornithine transport | 1 | Slc7a3 | 0.003882049 |
|  | GO:0001934 | positive regulation of protein phosphorylation | 3 | -,-,- | 0.004066893 |
|  | GO:0071479 | cellular response to ionizing radiation | 2 | Fignl1,Net1 | 0.004488191 |
|  | GO:1903243 | negative regulation of cardiac muscle hypertrophy in response to stress | 1 | Errfi1 | 0.004618355 |
|  | GO:2000299 | negative regulation of Rho-dependent protein serine/threonine kinase activity | 1 | Chordc1 | 0.004618355 |
|  | GO:0018205 | peptidyl-lysine modification | 1 | Atp7a | 0.004618355 |
|  | GO:0035491 | positive regulation of leukotriene production involved in inflammatory response | 1 | Serpine1 | 0.004618355 |
|  | GO:0042414 | epinephrine metabolic process | 1 | Atp7a | 0.004618355 |
|  | GO:0051542 | elastin biosynthetic process | 1 | Atp7a | 0.004618355 |
|  | GO:0061044 | negative regulation of vascular wound healing | 1 | Serpine1 | 0.004618355 |
|  | GO:1903748 | negative regulation of establishment of protein localization to mitochondrion | 1 | Hsph1 | 0.004618355 |
|  | GO:1903751 | negative regulation of intrinsic apoptotic signaling pathway in response to hydrogen peroxide | 1 | Hsph1 | 0.004618355 |
|  | GO:1903753 | negative regulation of p38MAPK cascade | 1 | Hsph1 | 0.004618355 |
|  | GO:0061050 | regulation of cell growth involved in cardiac muscle cell development | 1 | Col14a1 | 0.004620641 |
|  | GO:0007584 | response to nutrient | 3 | -,-,Cckar | 0.004625297 |
|  | GO:1900034 | regulation of cellular response to heat | 1 | Chordc1 | 0.005988728 |
|  | GO:0030199 | collagen fibril organization | 2 | Col14a1,Atp7a | 0.006428271 |
|  | GO:0006226 | dUMP biosynthetic process | 1 | - | 0.006779607 |
|  | GO:0046081 | dUTP catabolic process | 1 | - | 0.006779607 |
|  | GO:0005975 | carbohydrate metabolic process | 3 | -,-,- | 0.006805595 |
|  | GO:1902774 | late endosome to lysosome transport | 1 | - | 0.007113178 |
|  | GO:0044826 | viral genome integration into host DNA | 3 | -,-,- | 0.007180351 |
|  | GO:0075713 | establishment of integrated proviral latency | 3 | -,-,- | 0.007180351 |
|  | GO:0002377 | immunoglobulin production | 2 | -,- | 0.00756447 |
|  | GO:0034340 | response to type I interferon | 1 | Mx2 | 0.007564682 |
|  | GO:2000786 | positive regulation of autophagosome assembly | 1 | - | 0.007597713 |
|  | GO:0031017 | exocrine pancreas development | 1 | Ptf1a | 0.007602935 |
|  | GO:0046514 | ceramide catabolic process | 1 | Cel | 0.007609825 |
|  | GO:0061074 | regulation of neural retina development | 1 | Ptf1a | 0.007691471 |
|  | GO:0038094 | Fc-gamma receptor signaling pathway | 1 | Fcgrt | 0.007720676 |
|  | GO:0061113 | pancreas morphogenesis | 1 | Nr5a2 | 0.007749278 |
|  | GO:0048286 | lung alveolus development | 2 | Errfi1,Atp7a | 0.007775561 |
|  | GO:2000098 | negative regulation of smooth muscle cell-matrix adhesion | 1 | Serpine1 | 0.007951396 |
|  | GO:0042304 | regulation of fatty acid biosynthetic process | 1 | Pdk4 | 0.008912994 |
|  | GO:0070389 | chaperone cofactor-dependent protein refolding | 1 | Dnajb1 | 0.008971077 |
|  | GO:0006570 | tyrosine metabolic process | 1 | Atp7a | 0.00900844 |
|  | GO:0006568 | tryptophan metabolic process | 1 | Atp7a | 0.009052148 |
|  | GO:0042093 | T-helper cell differentiation | 1 | Atp7a | 0.009130901 |
|  | GO:0033687 | osteoblast proliferation | 1 | Fignl1 | 0.009167821 |
|  | GO:0001300 | chronological cell aging | 1 | Serpine1 | 0.009215676 |
|  | GO:0010041 | response to iron(III) ion | 1 | Atp7a | 0.009215676 |
|  | GO:0015679 | plasma membrane copper ion transport | 1 | Atp7a | 0.009215676 |
|  | GO:0031247 | actin rod assembly | 1 | Pdxp | 0.009215676 |
|  | GO:0010664 | negative regulation of striated muscle cell apoptotic process | 1 | Bag3 | 0.009220567 |
|  | GO:0015074 | DNA integration | 3 | -,-,- | 0.009271191 |
|  | GO:0046718 | viral entry into host cell | 3 | -,-,- | 0.009826261 |
|  | GO:0010273 | detoxification of copper ion | 1 | Atp7a | 0.01011675 |
|  | GO:0042144 | vacuole fusion, non-autophagic | 1 | - | 0.010968041 |
|  | GO:0006203 | dGTP catabolic process | 1 | Samhd1 | 0.011091689 |
|  | GO:0090274 | positive regulation of somatostatin secretion | 1 | Cckar | 0.011184085 |
|  | GO:0090403 | oxidative stress-induced premature senescence | 1 | Wnt16 | 0.011281029 |
|  | GO:0046061 | dATP catabolic process | 1 | Samhd1 | 0.011293924 |
|  | GO:0030299 | intestinal cholesterol absorption | 1 | Pnlip | 0.011535861 |
|  | GO:0015809 | arginine transport | 1 | Slc7a3 | 0.011596795 |
|  | GO:0097638 | L-arginine import across plasma membrane | 1 | Slc7a3 | 0.011601232 |
|  | GO:1903352 | L-ornithine transmembrane transport | 1 | Slc7a3 | 0.011601232 |
|  | GO:0021592 | fourth ventricle development | 1 | Myh10 | 0.012343104 |
|  | GO:0061073 | ciliary body morphogenesis | 1 | Tsku | 0.012407895 |
|  | GO:0045616 | regulation of keratinocyte differentiation | 1 | Errfi1 | 0.013367277 |
|  | GO:0002474 | antigen processing and presentation of peptide antigen via MHC class I | 2 | RT1-CE1,- | 0.013411548 |
|  | GO:0046320 | regulation of fatty acid oxidation | 1 | Pdk4 | 0.013473716 |
|  | GO:0042421 | norepinephrine biosynthetic process | 1 | Atp7a | 0.013635277 |
|  | GO:0048251 | elastic fiber assembly | 1 | Atp7a | 0.013791098 |
|  | GO:0090481 | pyrimidine nucleotide-sugar transmembrane transport | 1 | Slc35d1 | 0.013792057 |
|  | GO:0003229 | ventricular cardiac muscle tissue development | 1 | Col14a1 | 0.013794152 |
|  | GO:0042769 | DNA damage response, detection of DNA damage | 1 | Dnaja1 | 0.013877803 |
|  | GO:0043031 | negative regulation of macrophage activation | 1 | Tff2 | 0.014558361 |
|  | GO:0006930 | substrate-dependent cell migration, cell extension | 1 | Myh10 | 0.014796092 |
|  | GO:0002793 | positive regulation of peptide secretion | 1 | S100a8 | 0.014824422 |
|  | GO:0002023 | reduction of food intake in response to dietary excess | 1 | Cckar | 0.014848524 |
|  | GO:0009615 | response to virus | 2 | Mx2,Ifit3 | 0.015037655 |
|  | GO:0002082 | regulation of oxidative phosphorylation | 1 | Atp7a | 0.015051312 |
|  | GO:0046883 | regulation of hormone secretion | 1 | Cckar | 0.015128088 |
|  | GO:0043266 | regulation of potassium ion transport | 1 | Cckar | 0.015231645 |
|  | GO:0061469 | regulation of type B pancreatic cell proliferation | 1 | Errfi1 | 0.015710425 |
|  | GO:0048554 | positive regulation of metalloenzyme activity | 1 | Atp7a | 0.016200192 |
|  | GO:0070585 | protein localization to mitochondrion | 1 | Dnaja1 | 0.016323274 |
|  | GO:0021678 | third ventricle development | 1 | Myh10 | 0.016323631 |
|  | GO:0006914 | autophagy | 2 | S100a8,- | 0.016658743 |
|  | GO:0051918 | negative regulation of fibrinolysis | 1 | Serpine1 | 0.0171698 |
|  | GO:0097202 | activation of cysteine-type endopeptidase activity | 1 | Perp | 0.017526677 |
|  | GO:0021960 | anterior commissure morphogenesis | 1 | Tsku | 0.017590308 |
|  | GO:0010510 | regulation of acetyl-CoA biosynthetic process from pyruvate | 1 | Pdk4 | 0.017730706 |
|  | GO:0015677 | copper ion import | 1 | Atp7a | 0.017789129 |
|  | GO:0021860 | pyramidal neuron development | 1 | Atp7a | 0.01782996 |
|  | GO:0010757 | negative regulation of plasminogen activation | 1 | Serpine1 | 0.017853765 |
|  | GO:0019725 | cellular homeostasis | 1 | Rhot2 | 0.017865257 |
|  | GO:0060455 | negative regulation of gastric acid secretion | 1 | Tff2 | 0.017917656 |
|  | GO:0042536 | negative regulation of tumor necrosis factor biosynthetic process | 1 | Errfi1 | 0.017976849 |
|  | GO:2000757 | negative regulation of peptidyl-lysine acetylation | 1 | Klf15 | 0.018065919 |
|  | GO:0033629 | negative regulation of cell adhesion mediated by integrin | 1 | Serpine1 | 0.018185573 |
|  | GO:0097186 | amelogenesis | 1 | Perp | 0.018290302 |
|  | GO:0047497 | mitochondrion transport along microtubule | 1 | Rhot2 | 0.018679098 |
|  | GO:0051353 | positive regulation of oxidoreductase activity | 1 | Atp7a | 0.018716823 |
|  | GO:0006968 | cellular defense response | 1 | Pnliprp2 | 0.018798237 |
|  | GO:0021540 | corpus callosum morphogenesis | 1 | Tsku | 0.01896094 |
|  | GO:0006006 | glucose metabolic process | 2 | Ins1,Ins2 | 0.018981226 |
|  | GO:0035881 | amacrine cell differentiation | 1 | Ptf1a | 0.019189453 |
|  | GO:0045088 | regulation of innate immune response | 1 | Samhd1 | 0.019226063 |
|  | GO:0097345 | mitochondrial outer membrane permeabilization | 1 | Rhot2 | 0.020122877 |
|  | GO:0002934 | desmosome organization | 1 | Perp | 0.020613838 |
|  | GO:0002227 | innate immune response in mucosa | 1 | Pla2g1b | 0.020955078 |
|  | GO:0060428 | lung epithelium development | 1 | Errfi1 | 0.021430902 |
|  | GO:0030048 | actin filament-based movement | 1 | Myh10 | 0.021657742 |
|  | GO:0034058 | endosomal vesicle fusion | 1 | - | 0.022443457 |
|  | GO:0030262 | apoptotic nuclear changes | 1 | Dedd2 | 0.02250666 |
|  | GO:0030157 | pancreatic juice secretion | 1 | Cckar | 0.022508636 |
|  | GO:0001778 | plasma membrane repair | 1 | Myh10 | 0.022548209 |
|  | GO:0072112 | glomerular visceral epithelial cell differentiation | 1 | Klf15 | 0.022769437 |
|  | GO:0046889 | positive regulation of lipid biosynthetic process | 1 | Hsd17b13 | 0.022836982 |
|  | GO:0048699 | generation of neurons | 1 | Ptf1a | 0.023013906 |
|  | GO:0051451 | myoblast migration | 1 | Net1 | 0.023069211 |
|  | GO:0007589 | body fluid secretion | 1 | Tsc22d3 | 0.024030018 |
|  | GO:0048553 | negative regulation of metalloenzyme activity | 1 | Atp7a | 0.024071149 |
|  | GO:0071474 | cellular hyperosmotic response | 1 | Errfi1 | 0.024286102 |
|  | GO:0006878 | cellular copper ion homeostasis | 1 | Atp7a | 0.025887839 |
|  | GO:0006983 | ER overload response | 1 | Ins2 | 0.025914337 |
|  | GO:0046470 | phosphatidylcholine metabolic process | 1 | Pla2g1b | 0.02617051 |
|  | GO:0035542 | regulation of SNARE complex assembly | 1 | - | 0.026240641 |
|  | GO:0060426 | lung vasculature development | 1 | Errfi1 | 0.026516746 |
|  | GO:0009888 | tissue development | 1 | Ptf1a | 0.026595188 |
|  | GO:0042428 | serotonin metabolic process | 1 | Atp7a | 0.026796823 |
|  | GO:0035418 | protein localization to synapse | 1 | Mpp4 | 0.026825543 |
|  | GO:0042417 | dopamine metabolic process | 1 | Atp7a | 0.027272711 |
|  | GO:0019430 | removal of superoxide radicals | 1 | Atp7a | 0.027582664 |
|  | GO:0071260 | cellular response to mechanical stimulus | 2 | Bag3,Il13ra2 | 0.027837229 |
|  | GO:0043589 | skin morphogenesis | 1 | Errfi1 | 0.027985615 |
|  | GO:0007175 | negative regulation of epidermal growth factor-activated receptor activity | 1 | Errfi1 | 0.028212378 |
|  | GO:0006470 | protein dephosphorylation | 2 | Pdxp,Dusp8 | 0.028983574 |
|  | GO:0045124 | regulation of bone resorption | 1 | Pdk4 | 0.029133088 |
|  | GO:0002544 | chronic inflammatory response | 1 | S100a8 | 0.029496574 |
|  | GO:0002523 | leukocyte migration involved in inflammatory response | 1 | S100a8 | 0.029732836 |
|  | GO:0051223 | regulation of protein transport | 1 | Dnaja1 | 0.029805554 |
|  | GO:0030902 | hindbrain development | 1 | Ptf1a | 0.030189805 |
|  | GO:0060317 | cardiac epithelial to mesenchymal transition | 1 | Wnt16 | 0.030486705 |
|  | GO:0010469 | regulation of signaling receptor activity | 1 | Serpine1 | 0.031327573 |
|  | GO:0019731 | antibacterial humoral response | 1 | Pla2g1b | 0.032213314 |
|  | GO:0000027 | ribosomal large subunit assembly | 1 | - | 0.032290946 |
|  | GO:0010269 | response to selenium ion | 1 | - | 0.032907372 |
|  | GO:0043434 | response to peptide hormone | 2 | Reg1a,Ins1 | 0.033021199 |
|  | GO:0001696 | gastric acid secretion | 1 | Cckar | 0.033284586 |
|  | GO:0043508 | negative regulation of JUN kinase activity | 1 | Dnaja1 | 0.033492193 |
|  | GO:0090399 | replicative senescence | 1 | Wnt16 | 0.034054049 |
|  | GO:0055015 | ventricular cardiac muscle cell development | 1 | Myh10 | 0.034073496 |
|  | GO:0043616 | keratinocyte proliferation | 1 | Wnt16 | 0.034205185 |
|  | GO:0035457 | cellular response to interferon-alpha | 1 | Ifit3 | 0.034258505 |
|  | GO:0008206 | bile acid metabolic process | 1 | Nr5a2 | 0.03431783 |
|  | GO:0016236 | macroautophagy | 1 | - | 0.034322674 |
|  | GO:0030836 | positive regulation of actin filament depolymerization | 1 | Pdxp | 0.034383136 |
|  | GO:0032006 | regulation of TOR signaling | 1 | Slc7a3 | 0.034395139 |
|  | GO:0036120 | cellular response to platelet-derived growth factor stimulus | 1 | Errfi1 | 0.034568516 |
|  | GO:0071318 | cellular response to ATP | 1 | Pdxp | 0.034640887 |
|  | GO:0007097 | nuclear migration | 1 | Myh10 | 0.035648469 |
|  | GO:0021702 | cerebellar Purkinje cell differentiation | 1 | Atp7a | 0.035989596 |
|  | GO:0031953 | negative regulation of protein autophosphorylation | 1 | Errfi1 | 0.036174685 |
|  | GO:0055003 | cardiac myofibril assembly | 1 | Myh10 | 0.03667728 |
|  | GO:0045070 | positive regulation of viral genome replication | 1 | Nr5a2 | 0.037612524 |
|  | GO:0048384 | retinoic acid receptor signaling pathway | 1 | Ptf1a | 0.037694982 |
|  | GO:0043065 | positive regulation of apoptotic process | 3 | -,Dnaja1,Net1 | 0.037729434 |
|  | GO:0014898 | cardiac muscle hypertrophy in response to stress | 1 | Klf15 | 0.03782659 |
|  | GO:0007088 | regulation of mitotic nuclear division | 1 | Pdxp | 0.037881731 |
|  | GO:1900006 | positive regulation of dendrite development | 1 | Reg1a | 0.038064055 |
|  | GO:0032966 | negative regulation of collagen biosynthetic process | 1 | Errfi1 | 0.038744538 |
|  | GO:0021680 | cerebellar Purkinje cell layer development | 1 | Myh10 | 0.039600135 |
|  | GO:0030206 | chondroitin sulfate biosynthetic process | 1 | Slc35d1 | 0.039715369 |
|  | GO:0048599 | oocyte development | 1 | Ybx2 | 0.039904446 |
|  | GO:0042572 | retinol metabolic process | 1 | Cel | 0.040282908 |
|  | GO:0032691 | negative regulation of interleukin-1 beta production | 1 | Errfi1 | 0.040283847 |
|  | GO:0010468 | regulation of gene expression | 2 | Tsku,Atp7a | 0.040338291 |
|  | GO:0002526 | acute inflammatory response | 1 | S100a8 | 0.040374863 |
|  | GO:0048193 | Golgi vesicle transport | 1 | - | 0.040690292 |
|  | GO:1901652 | response to peptide | 1 | Cckar | 0.041385422 |
|  | GO:0007494 | midgut development | 1 | Reg1a | 0.041668412 |
|  | GO:0060416 | response to growth hormone | 1 | Cacybp | 0.042511796 |
|  | GO:2000811 | negative regulation of anoikis | 1 | Pdk4 | 0.042757906 |
|  | GO:1901653 | cellular response to peptide | 1 | Klf15 | 0.043559288 |
|  | GO:0090026 | positive regulation of monocyte chemotaxis | 1 | Serpine1 | 0.043903196 |
|  | GO:0007512 | adult heart development | 1 | Myh10 | 0.043961988 |
|  | GO:0050732 | negative regulation of peptidyl-tyrosine phosphorylation | 1 | Errfi1 | 0.044224486 |
|  | GO:0045055 | regulated exocytosis | 1 | - | 0.04444419 |
|  | GO:0035871 | protein K11-linked deubiquitination | 1 | Otub2 | 0.044786545 |
|  | GO:0048663 | neuron fate commitment | 1 | Ptf1a | 0.044894269 |
|  | GO:0042059 | negative regulation of epidermal growth factor receptor signaling pathway | 1 | Errfi1 | 0.044943618 |
|  | GO:0007520 | myoblast fusion | 1 | Ins2 | 0.045079533 |
|  | GO:0031000 | response to caffeine | 1 | - | 0.045129526 |
|  | GO:0010033 | response to organic substance | 2 | Ins1,- | 0.048212622 |
|  | GO:0010906 | regulation of glucose metabolic process | 1 | Pdk4 | 0.048303532 |
|  | GO:0061436 | establishment of skin barrier | 1 | Cela2a | 0.04868995 |
|  | GO:0014912 | negative regulation of smooth muscle cell migration | 1 | Serpine1 | 0.049176877 |
|  | GO:0010824 | regulation of centrosome duplication | 1 | Chordc1 | 0.049741854 |
| CC | GO:0005615 | extracellular space | 18 | Pla2g1b,Tff2,Wnt16,Reg1a,Serpini2,Cel,Cpa1,S100a8,Ins1,LOC312273,-,Ins2,-,-,-,-,-,- | 1.72E-09 |
|  | GO:0097226 | sperm mitochondrial sheath | 3 | -,-,- | 3.00E-08 |
|  | GO:0097524 | sperm plasma membrane | 3 | -,-,- | 3.00E-08 |
|  | GO:0005576 | extracellular region | 12 | Hsd17b13,-,Cela2a,Gp2,Pnlip,Pnliprp1,Pnliprp2,Cpa2,-,-,-,- | 9.93E-08 |
|  | GO:0020002 | host cell plasma membrane | 7 | -,-,-,-,-,-,- | 2.54E-07 |
|  | GO:0044294 | dendritic growth cone | 3 | -,-,- | 1.75E-06 |
|  | GO:0062023 | collagen-containing extracellular matrix | 3 | -,-,- | 1.20E-05 |
|  | GO:0055036 | virion membrane | 4 | -,-,-,- | 2.29E-05 |
|  | GO:0019031 | viral envelope | 4 | -,-,-,- | 2.55E-05 |
|  | GO:0044295 | axonal growth cone | 3 | -,-,- | 4.29E-05 |
|  | GO:0031526 | brush border membrane | 4 | Atp7a,-,-,- | 4.40E-05 |
|  | GO:0036126 | sperm flagellum | 3 | -,-,- | 4.82E-05 |
|  | GO:0032991 | protein-containing complex | 3 | -,-,- | 5.95E-05 |
|  | GO:0043005 | neuron projection | 6 | Cacybp,Bag3,Atp7a,-,-,- | 0.000309774 |
|  | GO:0042589 | zymogen granule membrane | 2 | Zg16,Cuzd1 | 0.000375104 |
|  | GO:0042588 | zymogen granule | 2 | Reg1a,Cel | 0.000507768 |
|  | GO:0030141 | secretory granule | 3 | Pla2g1b,Ins1,Ins2 | 0.001489317 |
|  | GO:0042470 | melanosome | 3 | -,-,- | 0.001796548 |
|  | GO:0044185 | host cell late endosome membrane | 3 | -,-,- | 0.00215748 |
|  | GO:0072494 | host multivesicular body | 3 | -,-,- | 0.002293116 |
|  | GO:0016323 | basolateral plasma membrane | 4 | Atp7a,-,-,- | 0.002475534 |
|  | GO:0043209 | myelin sheath | 4 | Stip1,-,-,- | 0.00247872 |
|  | GO:0005829 | cytosol | 12 | Hsph1,Dnaja1,Pdxp,Dnaja4,Bag3,Dnajb1,Hspa1l,Errfi1,-,-,-,- | 0.00294622 |
|  | GO:0019814 | immunoglobulin complex | 2 | -,- | 0.004219925 |
|  | GO:0097513 | myosin II filament | 1 | Myh10 | 0.004618363 |
|  | GO:0019013 | viral nucleocapsid | 3 | -,-,- | 0.004710554 |
|  | GO:0032154 | cleavage furrow | 2 | Myh10,Pdxp | 0.004971641 |
|  | GO:0042612 | MHC class I protein complex | 2 | RT1-CE1,- | 0.009010098 |
|  | GO:0070062 | extracellular exosome | 14 | Hsph1,Serpine1,Cacybp,Myh10,LOC498222,Fignl1,Galnt16,Dnaja1,Pdxp,Rhot2,Dnajb1,Col14a1,-,Calml3 | 0.009725133 |
|  | GO:0043025 | neuronal cell body | 5 | Myh10,Atp7a,-,-,- | 0.010308393 |
|  | GO:0060205 | cytoplasmic membrane-bounded vesicle lumen | 1 | Zg16 | 0.011225046 |
|  | GO:0070938 | contractile ring | 1 | Pdxp | 0.013779758 |
|  | GO:0030123 | AP-3 adaptor complex | 1 | - | 0.014808163 |
|  | GO:0030877 | beta-catenin destruction complex | 1 | Cacybp | 0.017245963 |
|  | GO:0071439 | clathrin complex | 1 | - | 0.018344417 |
|  | GO:0016528 | sarcoplasm | 1 | Flnc | 0.021092418 |
|  | GO:0005634 | nucleus | 18 | Foxo3,-,Cacybp,Myh10,Fignl1,Dnaja1,Cdkn2c,Otub2,Net1,Klf15,Dnajb1,Dusp8,-,Tsc22d3,Errfi1,-,-,- | 0.021490179 |
|  | GO:0016012 | sarcoglycan complex | 1 | Sgca | 0.021814771 |
|  | GO:0005796 | Golgi lumen | 1 | Zg16 | 0.02208933 |
|  | GO:0022625 | cytosolic large ribosomal subunit | 2 | -,- | 0.022337705 |
|  | GO:0016460 | myosin II complex | 1 | Myh10 | 0.023073659 |
|  | GO:0044297 | cell body | 2 | Cacybp,Hspa1l | 0.023168905 |
|  | GO:0005732 | small nucleolar ribonucleoprotein complex | 1 | Ins2 | 0.025216295 |
|  | GO:0031932 | TORC2 complex | 1 | - | 0.026326725 |
|  | GO:0005929 | cilium | 2 | Pkhd1l1,Evc2 | 0.026947709 |
|  | GO:0072562 | blood microparticle | 2 | Hspa1l,- | 0.030315324 |
|  | GO:0005770 | late endosome | 2 | -,- | 0.031049574 |
|  | GO:0042383 | sarcolemma | 2 | Sgca,Flnc | 0.031649295 |
|  | GO:0030018 | Z disc | 2 | Flnc,Bag3 | 0.035969702 |
|  | GO:0045178 | basal part of cell | 1 | Reg1a | 0.037616237 |
|  | GO:0048471 | perinuclear region of cytoplasm | 5 | Dnaja1,Atp7a,-,-,- | 0.038122913 |
|  | GO:0005641 | nuclear envelope lumen | 1 | Cacybp | 0.041401952 |
|  | GO:0030897 | HOPS complex | 1 | - | 0.044945376 |
|  | GO:0002199 | zona pellucida receptor complex | 1 | Hspa1l | 0.046415992 |
|  | GO:0030140 | trans-Golgi network transport vesicle | 1 | Atp7a | 0.047564694 |
| MF | GO:0004252 | serine-type endopeptidase activity | 11 | LOC312273,Prss3b,Cela2a,Ctrc,Ctrb1,Psmb10; Ctrl,Cela3b,-,-,-,- | 2.57E-11 |
|  | GO:0004806 | triglyceride lipase activity | 4 | Cel,Pnlip,Pnliprp1,Pnliprp2 | 2.42E-08 |
|  | GO:1990782 | protein tyrosine kinase binding | 3 | -,-,- | 3.00E-08 |
|  | GO:0004556 | alpha-amylase activity | 3 | -,-,- | 1.61E-07 |
|  | GO:0051082 | unfolded protein binding | 5 | Dnaja1,Dnaja4,-,-,- | 1.72E-07 |
|  | GO:0051022 | Rho GDP-dissociation inhibitor binding | 3 | -,-,- | 3.95E-07 |
|  | GO:0002135 | CTP binding | 3 | -,-,- | 4.55E-07 |
|  | GO:0030911 | TPR domain binding | 3 | -,-,- | 4.66E-07 |
|  | GO:0043169 | cation binding | 3 | -,-,- | 8.86E-07 |
|  | GO:0017098 | sulfonylurea receptor binding | 3 | -,-,- | 1.02E-06 |
|  | GO:0002134 | UTP binding | 3 | -,-,- | 1.02E-06 |
|  | GO:0070182 | DNA polymerase binding | 3 | -,-,- | 1.38E-06 |
|  | GO:0030235 | nitric-oxide synthase regulator activity | 3 | -,-,- | 1.55E-06 |
|  | GO:0032564 | dATP binding | 3 | -,-,- | 1.87E-06 |
|  | GO:0097718 | disordered domain specific binding | 3 | -,-,- | 3.98E-06 |
|  | GO:0048156 | tau protein binding | 3 | -,-,- | 1.12E-05 |
|  | GO:0097110 | scaffold protein binding | 3 | -,-,- | 1.89E-05 |
|  | GO:0051020 | GTPase binding | 3 | -,-,- | 2.25E-05 |
|  | GO:0103025 | alpha-amylase activity (releasing maltohexaose) | 2 | -,- | 3.32E-05 |
|  | GO:0019903 | protein phosphatase binding | 3 | -,-,- | 6.20E-05 |
|  | GO:0004181 | metallocarboxypeptidase activity | 3 | Cpa1,Cpa2,Cpb1 | 6.80E-05 |
|  | GO:0031404 | chloride ion binding | 2 | -,- | 0.000124306 |
|  | GO:0004867 | serine-type endopeptidase inhibitor activity | 4 | Serpini2,-,-,- | 0.000138194 |
|  | GO:0044325 | ion channel binding | 3 | -,-,- | 0.000294493 |
|  | GO:0042826 | histone deacetylase binding | 3 | -,-,- | 0.000792482 |
|  | GO:0016829 | lyase activity | 2 | Cth,- | 0.001084894 |
|  | GO:0031625 | ubiquitin protein ligase binding | 3 | -,-,- | 0.00115834 |
|  | GO:0039660 | structural constituent of virion | 3 | -,-,- | 0.002840768 |
|  | GO:0050253 | retinyl-palmitate esterase activity | 1 | Cel | 0.003742205 |
|  | GO:0004951 | cholecystokinin receptor activity | 1 | Cckar | 0.003818983 |
|  | GO:0008238 | exopeptidase activity | 1 | Cpa1 | 0.003839642 |
|  | GO:0030881 | beta-2-microglobulin binding | 1 | Fcgrt | 0.003882061 |
|  | GO:0008832 | dGTPase activity | 1 | Samhd1 | 0.003882061 |
|  | GO:0032567 | dGTP binding | 1 | Samhd1 | 0.003882061 |
|  | GO:0002020 | protease binding | 2 | Ins1,Ins2 | 0.003965634 |
|  | GO:0042802 | identical protein binding | 3 | -,-,- | 0.00621729 |
|  | GO:0070180 | large ribosomal subunit rRNA binding | 1 | - | 0.006870178 |
|  | GO:0004522 | ribonuclease A activity | 1 | - | 0.007008185 |
|  | GO:0051087 | chaperone binding | 2 | Dnaja1,Dnaja4 | 0.007258951 |
|  | GO:0019770 | IgG receptor activity | 1 | Fcgrt | 0.007720676 |
|  | GO:0003729 | mRNA binding | 3 | -,-,- | 0.008268989 |
|  | GO:0016532 | superoxide dismutase copper chaperone activity | 1 | Atp7a | 0.00828418 |
|  | GO:0031267 | small GTPase binding | 1 | Errfi1 | 0.009196913 |
|  | GO:0004008 | copper-exporting ATPase activity | 1 | Atp7a | 0.009298413 |
|  | GO:0004190 | aspartic-type endopeptidase activity | 3 | -,-,- | 0.009684299 |
|  | GO:0004523 | RNA-DNA hybrid ribonuclease activity | 3 | -,-,- | 0.009823666 |
|  | GO:0004771 | sterol esterase activity | 1 | Cel | 0.011436474 |
|  | GO:0004032 | alditol:NADP+ 1-oxidoreductase activity | 1 | Akr7a3 | 0.011526392 |
|  | GO:0000064 | L-ornithine transmembrane transporter activity | 1 | Slc7a3 | 0.011601232 |
|  | GO:0042277 | peptide binding | 2 | Cckar,- | 0.011610051 |
|  | GO:0003887 | DNA-directed DNA polymerase activity | 3 | -,-,- | 0.013519633 |
|  | GO:0015165 | pyrimidine nucleotide-sugar transmembrane transporter activity | 1 | Slc35d1 | 0.013792057 |
|  | GO:0003823 | antigen binding | 2 | -,- | 0.014114639 |
|  | GO:0004743 | pyruvate kinase activity | 1 | LOC100362738 | 0.015329722 |
|  | GO:0015174 | basic amino acid transmembrane transporter activity | 1 | Slc7a3 | 0.015432708 |
|  | GO:0015181 | arginine transmembrane transporter activity | 1 | Slc7a3 | 0.015438933 |
|  | GO:0015189 | L-lysine transmembrane transporter activity | 1 | Slc7a3 | 0.015438933 |
|  | GO:0004033 | aldo-keto reductase (NADP) activity | 1 | Akr7a3 | 0.016662712 |
|  | GO:0030506 | ankyrin binding | 1 | Flnc | 0.01922067 |
|  | GO:0042605 | peptide antigen binding | 2 | RT1-CE1,- | 0.019293413 |
|  | GO:0016887 | ATPase activity | 3 | -,-,- | 0.019503246 |
|  | GO:0005375 | copper ion transmembrane transporter activity | 1 | Atp7a | 0.020093258 |
|  | GO:0050544 | arachidonic acid binding | 1 | S100a8 | 0.022263078 |
|  | GO:0047498 | calcium-dependent phospholipase A2 activity | 1 | Pla2g1b | 0.022436477 |
|  | GO:0004170 | dUTP diphosphatase activity | 1 | - | 0.022586924 |
|  | GO:0043208 | glycosphingolipid binding | 1 | Cel | 0.022636441 |
|  | GO:0004622 | lysophospholipase activity | 1 | Cel | 0.022904868 |
|  | GO:0003823 | antigen binding | 2 | Fcgrt,- | 0.024917943 |
|  | GO:0016209 | antioxidant activity | 1 | S100a8 | 0.02564927 |
|  | GO:0005158 | insulin receptor binding | 1 | Ins1 | 0.026414779 |
|  | GO:0003956 | NAD(P)+-protein-arginine ADP-ribosyltransferase activity | 1 | Art4 | 0.02657173 |
|  | GO:0030955 | potassium ion binding | 1 | LOC100362738 | 0.026649319 |
|  | GO:0008568 | microtubule-severing ATPase activity | 1 | Fignl1 | 0.027244671 |
|  | GO:0005524 | ATP binding | 11 | Hsph1,Myh10,Fignl1,Dnaja1,Pdk4,Dnaja4,Hspa1l,Atp7a,-,-,- | 0.029153841 |
|  | GO:0008239 | dipeptidyl-peptidase activity | 1 | Dpep3 | 0.030807814 |
|  | GO:0008235 | metalloexopeptidase activity | 1 | Dpep3 | 0.030938859 |
|  | GO:0030898 | actin-dependent ATPase activity | 1 | Myh10 | 0.033302628 |
|  | GO:0004861 | cyclin-dependent protein serine/threonine kinase inhibitor activity | 1 | Cdkn2c | 0.033735417 |
|  | GO:0030247 | polysaccharide binding | 1 | Sbspon | 0.035900352 |
|  | GO:0042803 | protein homodimerization activity | 3 | -,-,- | 0.037362185 |
|  | GO:0000146 | microfilament motor activity | 1 | Myh10 | 0.037952171 |
|  | GO:0003964 | RNA-directed DNA polymerase activity | 3 | -,-,- | 0.040521687 |
|  | GO:0001671 | ATPase activator activity | 1 | Dnajb1 | 0.043714832 |
|  | GO:0016805 | dipeptidase activity | 1 | Dpep3 | 0.044795762 |
|  | GO:0030544 | Hsp70 protein binding | 1 | Dnaja1 | 0.048250088 |
