# Supplementary material for: Immune Responses to Multi-Frequencies of 1.5 GHz and 4.3 GHz Microwave Exposure in Rats: Transcriptomic and Proteomic Analysis
Source: Int J Mol Sci. 2022 Jun 22;23(13):6949. doi: 10.3390/ijms23136949 (PMC9266614; doi:10.3390/ijms23136949)
Supplement: Supplementary file 1 [file ijms-23-06949-s001.zip › Supplementary Table S3.docx]

**Supplementary Table S3 GO analysis of DEPs between LC10 exposure and Sham exposure in peripheral blood**

| **GO** | **ID** | **Categories** | **Number of DEPs** | **Name of proteins** | **P value** |
| --- | --- | --- | --- | --- | --- |
| BP | GO:0010498 | proteasomal protein catabolic process | 10 | Psma2,Psma1,Psma3,Psma4,Psmb8,Psmb3,Psmb2,Vcp,Psma6,Psmb10 | 0.00028 |
|  | GO:0010499 | proteasomal ubiquitin-independent protein catabolic process | 9 | Psma2,Psma1,Psma3,Psma4,Psmb8,Psmb3,Psmb2,Psma6,Psmb10 | 0.000717 |
|  | GO:0043161 | proteasome-mediated ubiquitin-dependent protein catabolic process | 11 | Psma2,Psma1,Psma3,Psma4,Psmb8,Psmb3,Psmb2,Vcp,Psma6,Psmb10,Psma5 | 0.003815 |
|  | GO:0021766 | hippocampus development | 5 | Cfl1,Hnrnpk,Ywhae,Ran,Kif5b | 0.006372 |
|  | GO:0006890 | retrograde vesicle-mediated transport, Golgi to endoplasmic reticulum | 5 | Rab6a,Copz1,Copb2,Copb1,Arf5 | 0.006372 |
|  | GO:0008360 | regulation of cell shape | 7 | Rhof,Diaph1,F2,Rlc-a,Rhoa,Wdr1,Coro1a | 0.01404 |
|  | GO:0032092 | positive regulation of protein binding | 4 | Add1,Stk3,Lrp1,Hsp90ab1 | 0.017623 |
|  | GO:0007339 | binding of sperm to zona pellucida | 4 | Cct8,Tcp1,Cct2,Cct3 | 0.017623 |
|  | GO:0090307 | mitotic spindle assembly | 4 | Flna,Kif2a,Kpnb1,Rhoa | 0.017623 |
|  | GO:0007010 | cytoskeleton organization | 4 | Diaph1,Cfl1,Dpysl2,Capzb | 0.017623 |
|  | GO:0030334 | regulation of cell migration | 4 | Vcl,Rhof,Kif2a,Rhoa | 0.017623 |
|  | GO:0006888 | endoplasmic reticulum to Golgi vesicle-mediated transport | 5 | LOC100363782,Copb2,Copb1,Uso1,Vcp | 0.026747 |
|  | GO:1901998 | toxin transport | 5 | Copz1,Cct8,Tcp1,Cct2,Cct3 | 0.026747 |
|  | GO:0051017 | actin filament bundle assembly | 5 | Add1,Rhof,Coro1b,Rhoa,Actn1 | 0.026747 |
|  | GO:0051016 | barbed-end actin filament capping | 5 | Add1,Capza1,Capza2,Twf1,Capzb | 0.026747 |
|  | GO:0030036 | actin cytoskeleton organization | 7 | Specc1,Capza1,Inf2,Diaph1,Rhoa,Capza2,Capzb | 0.032312 |
|  | GO:1901224 | positive regulation of NIK/NF-kappaB signaling | 3 | Calr,Rhoa,Ilk | 0.048591 |
|  | GO:0030032 | lamellipodium assembly | 3 | Vcl,Parvb,Cyfip1 | 0.048591 |
|  | GO:0050919 | negative chemotaxis | 3 | Slit1,Nrp2,Rhoa | 0.048591 |
|  | GO:0061025 | membrane fusion | 3 | Dnm1l,Uso1,Napa | 0.048591 |
|  | GO:0019835 | cytolysis | 3 | Lyz2,C8b,C6 | 0.048591 |
|  | GO:1903078 | positive regulation of protein localization to plasma membrane | 3 | Sptbn1,Lrp1,Kif5b | 0.048591 |
|  | GO:0007264 | small GTPase mediated signal transduction | 3 | Rac1,Arhgap1,Rasgrp2 | 0.048591 |
|  | GO:0030042 | actin filament depolymerization | 3 | Twf1,Wdr1,Dstn | 0.048591 |
|  | GO:0051131 | chaperone-mediated protein complex assembly | 3 | Hspa4,Clu,Cct2 | 0.048591 |
|  | GO:0007411 | axon guidance | 3 | Cyfip1,Nrp2,Dpysl2 | 0.048591 |
|  | GO:0051279 | regulation of release of sequestered calcium ion into cytosol | 3 | Ubash3b,Diaph1,Coro1a | 0.048591 |
|  | GO:0043297 | apical junction assembly | 3 | Vcl,Rhoa,Wdr1 | 0.048591 |
|  | GO:0030836 | positive regulation of actin filament depolymerization | 3 | Cfl1,Wdr1,Dstn | 0.048591 |
|  | GO:0048675 | axon extension | 3 | Vcl,Cyfip1,Hsp90aa1 | 0.048591 |
|  | GO:0006886 | intracellular protein transport | 10 | Ap1s1,Copz1,Sytl4,Copb2,Copb1,Uso1,Napa,Arf5,Ap1m1,Sar1a | 0.049268 |
| CC | GO:0005829 | cytosol | 64 | Pdia6,Fhod1,Sars,Txnl1,Masp1,Rab6a,Tpm3,Ap1s1,Flna,Dctn3,Mob1b,Ipo7,Alox12,Hspa4,Prkca,Kpnb1,C9,Sytl4,Unc13d,Clu,Coro1b,Copb2,Pafah1b2,Dnm1l,Gnai2,G6pdx,Ckb,Pkm,Prkar2b,Calr,Gucy1b1,Psma4,Copb1,Hsp90ab1,Uso1,Vcp,Dpysl2,St13,Rhoa,Hnrnpk,Ppp1ca,Ppp1cb,Ywhae,Ran,Actg1,Hsp90aa1,Kif5b,Twf1,Uba1,Rap1b,Pde3a,Ppp6c,Cavin2,Eef1d,Cops3,Prune1,Psmb9,Psma5,Coro1a,Skap2,Ilk,Glrx3,Actn1,Rasgrp2 | 7.22E-09 |
|  | GO:0005737 | cytoplasm | 68 | Cmpk1,Add1,Mylk,Psma7,Mpo,Stk3,Ctps1,Parvb,Rhof,Ipo5,Cyfip1,Snd1,Myh14,Alox12,Diaph1,Tubb4b,Psmb4,Tubb1,Pafah1b2,Dnm1l,Fgg,Gnai2,Pkm,Prkar2b,Psma2,Calr,Psma1,Psma3,Psma4,Psmb8,Hsp90ab1,Psmb3,Psmb2,Cfl1,Vcp,Psma6,Rhoa,Ywhag,Ppp1ca,Ppp1cb,Ywhae,Ran,Pfn1,Ywhaq,Tuba1a,Ywhah,Hsp90aa1,Arf5,Tubb2a,Kif5b,Psmb10,Tubb6,Twf1,Uba1,Tuba4a,Tpm3,Npl,Cavin2,Eef1g,Tuba8,Prune1,Clic1,Eif4a1,Psmb1,Ehd3,Skap2,Ilk,Actn1 | 9.85E-06 |
|  | GO:0005839 | proteasome core complex | 12 | Psmb4,Psma2,Psma1,Psma3,Psma4,Psmb8,Psmb3,Psmb2,Psma6,Psmb10,Psmb9,Psmb1 | 9.70E-05 |
|  | GO:0005634 | nucleus | 43 | Cmpk1,Fhod1,Psma7,Mpo,Pfkm,Flna,Diaph1,Clu,Cavin1,Psmb4,Gnai2,G6pdx,Ckb,Pkm,Psma2,Calr,Psma1,Psma3,Psma4,Psmb8,Hsp90ab1,Psmb3,Psmb2,Vcp,Psma6,Rhoa,Hnrnpk,Ppp1ca,Ppp1cb,Ywhae,Ran,Pfn1,Hsp90aa1,Psmb10,Uba1,Eef1g,Prune1,Clic1,Psmb9,Psma5,Psmb1,Glrx3,Actn1 | 0.000105 |
|  | GO:0005654 | nucleoplasm | 20 | Utrn,Masp1,Lnpk,Cct8,Ipo7,Kpnb1,Sytl4,Pip4k2b,Gnai2,Psma1,Psmb2,Vcp,Hnrnpk,Ppp1ca,Ppp1cb,Uba1,Cavin2,Cops3,Skap2,Ilk | 0.000106 |
|  | GO:0005874 | microtubule | 15 | Dnm2,Cct8,Tubb4b,0,Tubb1,Dnm1l,Tcp1,Tuba1a,Tubb2a,Kif5b,Tubb6,Tuba4a,Cct2,Tuba8,Cct3 | 0.000476 |
|  | GO:0019773 | proteasome core complex, alpha-subunit complex | 7 | Psma7,Psma2,Psma1,Psma3,Psma4,Psma6,Psma5 | 0.000532 |
|  | GO:0005579 | membrane attack complex | 6 | C5,C7,C8a,C9,C8b,C6 | 0.001576 |
|  | GO:0098794 | postsynapse | 9 | Psma7,Sptbn1,Flna,Dnm1l,Prkar2b,Napa,Hnrnpk,Ppp1ca,Pfn1 | 0.001613 |
|  | GO:0005730 | nucleolus | 7 | Sptbn1,Flna,Dctn3,Mob1b,Kif2a,Ppp1ca,Ppp1cb | 0.003002 |
|  | GO:0098978 | glutamatergic synapse | 17 | Sptbn1,Flna,Sparcl1,Dlgap4,Erbin,Nrp2,Prkar2b,Gucy1b1,Cfl1,Vcp,Napa,Rhoa,Hnrnpk,Ppp1ca,Ywhae,Pfn1,Coro1a | 0.00321 |
|  | GO:0005911 | cell-cell junction | 8 | Flna,Erbin,Cfl1,Twf1,Wdr1,Rap1b,Coro1a,Ilk | 0.003972 |
|  | GO:0005884 | actin filament | 6 | Flna,Coro1b,Tpm4,Twf1,Tpm3,Coro1a | 0.007844 |
|  | GO:0030054 | cell junction | 7 | Utrn,Erbin,Dnm1l,C4,Hnrnpk,Bin2,Actn1 | 0.009557 |
|  | GO:0001725 | stress fiber | 7 | Fhod1,Mylk,Tpm3,Myl9,Myh14,Coro1b,Rlc-a | 0.009557 |
|  | GO:0045202 | synapse | 10 | Cyfip1,Clu,Erbin,C4,Psma3,Ywhag,Ywhae,Pfn1,Ywhaq,Ywhah | 0.010012 |
|  | GO:0030027 | lamellipodium | 8 | Mylk,Parvb,Cyfip1,Coro1b,Cfl1,Rhoa,Coro1a,Ilk | 0.010262 |
|  | GO:0005856 | cytoskeleton | 8 | Add3,Clu,Tln1,Dpysl2,Rhoa,Pfn1,Actg1,Actn1 | 0.010262 |
|  | GO:0043197 | dendritic spine | 8 | Cyfip1,Prkar2b,Cfl1,Rhoa,Hnrnpk,Ppp1ca,Capzb,Actn1 | 0.010262 |
|  | GO:0002199 | zona pellucida receptor complex | 4 | Cct8,Tcp1,Cct2,Cct3 | 0.013721 |
|  | GO:0008290 | F-actin capping protein complex | 4 | Add1,Capza1,Capza2,Capzb | 0.013721 |
|  | GO:0044295 | axonal growth cone | 4 | Cyfip1,Lrp1,Hsp90aa1,Kif5b | 0.013721 |
|  | GO:0032587 | ruffle membrane | 4 | Diaph1,Cfl1,Rhoa,Twf1 | 0.013721 |
|  | GO:0044297 | cell body | 5 | Cct8,Gnai2,Tcp1,Cct2,Cct3 | 0.020069 |
|  | GO:0043025 | neuronal cell body | 11 | Flna,Cyfip1,Lrp1,Ckb,Prkar2b,Cfl1,Dpysl2,Ppp1ca,Hsp90aa1,Capzb,Ilk | 0.028361 |
|  | GO:0016460 | myosin II complex | 3 | Myl9,Myh14,Rlc-a | 0.040286 |
|  | GO:0001650 | fibrillar center | 3 | Pafah1b2,Uso1,Eef1d | 0.040286 |
|  | GO:0005635 | nuclear envelope | 3 | Ipo7,Calr,Clic1 | 0.040286 |
|  | GO:0031941 | filamentous actin | 3 | Specc1,Flna,Actg1 | 0.040286 |
|  | GO:0030016 | myofibril | 3 | Psma6,Actg1,Twf1 | 0.040286 |
|  | GO:0019774 | proteasome core complex, beta-subunit complex | 3 | Psmb8,Psmb3,Psmb2 | 0.040286 |
|  | GO:0071782 | endoplasmic reticulum tubular network | 3 | Lnpk,Kpnb1,Rab10 | 0.040286 |
|  | GO:0043198 | dendritic shaft | 3 | Flna,Prkar2b,Ilk | 0.040286 |
|  | GO:0030425 | dendrite | 7 | Lrp1,Gnai2,Ckb,C4,Prkar2b,Dpysl2,Glrx3 | 0.04416 |
|  | GO:0002102 | podosome | 6 | Vcl,Fermt3,Tpm4,Hnrnpk,Wdr1,Bin2 | 0.047999 |
|  | GO:0043195 | terminal bouton | 4 | Ap1s1,Cyfip1,Napa,Ilk | 0.049974 |
|  | GO:0030018 | Z disc | 4 | Myl9,Rlc-a,Glrx3,Actn1 | 0.049974 |
|  | GO:0005903 | brush border | 4 | Myh14,Dnm1l,Capza2,Tpm3 | 0.049974 |
|  | GO:0005832 | chaperonin-containing T-complex | 4 | Cct8,Tcp1,Cct2,Cct3 | 0.049974 |
| MF | GO:0051015 | actin filament binding | 19 | Add1,Utrn,Vcl,Capza1,Flna,Myh14,Myh9,Tln1,Coro1b,Tpm4,Cfl1,Capza2,Twf1,Wdr1,Capzb,Tpm3,Dstn,Coro1a,Actn1 | 4.28E-06 |
|  | GO:0005525 | GTP binding | 25 | Dnm2,44085,Rac1,Rab6a,Rhof,LOC100363782,Tubb4b,Tubb1,Dnm1l,Gnai2,Gucy1b1,Hsp90ab1,Rhoa,Ran,Tuba1a,Hsp90aa1,Arf5,Tubb2a,Tubb6,Rab10,Tuba4a,Rap1b,Sar1a,Tuba8,Ehd3 | 1.27E-05 |
|  | GO:0004298 | threonine-type endopeptidase activity | 14 | Psma7,Psmb4,Psma2,Psma1,Psma3,Psma4,Psmb8,Psmb3,Psmb2,Psma6,Psmb10,Psmb9,Psma5,Psmb1 | 2.68E-05 |
|  | GO:0044877 | protein-containing complex binding | 17 | Sptbn1,Flna,C8g,C8a,Clu,Tln1,Lrp1,Dnm1l,Vcp,St13,Napa,C8b,Ppp1ca,Ywhae,Ran,Ywhaz,Rap1b | 0.000448 |
|  | GO:0019904 | protein domain specific binding | 17 | Fhod1,Rab6a,Kpnb1,Prkar2b,Vcp,St13,Rhoa,Hnrnpk,Ywhag,Ywhae,Ran,Ywhaz,Ywhaq,Tuba1a,Ywhah,Ilk,Actn1 | 0.000448 |
|  | GO:0005524 | ATP binding | 30 | Cmpk1,Sars,Mylk,Yes1,Pfkm,Stk3,Ctps1,Cct8,Myh14,Taok3,Hspa4,Prkca,Kif2a,Myh9,Pip4k2b,Ckb,Pkm,Tcp1,Hsp90ab1,Vcp,Actg1,Hsp90aa1,Kif5b,Twf1,Uba1,Cct2,Eif4a1,Cct3,Ehd3,Ilk | 0.001151 |
|  | GO:0031625 | ubiquitin protein ligase binding | 13 | Vcl,Ubash3b,Clu,Dnm1l,Ckb,Prkar2b,Calr,Psma3,Tcp1,Vcp,Ywhae,Actg1,Cct2 | 0.001739 |
|  | GO:0003924 | GTPase activity | 18 | Dnm2,Rac1,Rab6a,Rhof,LOC100363782,Tubb4b,Tubb1,Dnm1l,Gnai2,Rhoa,Ran,Tuba1a,Tubb2a,Tubb6,Rab10,Tuba4a,Rap1b,Tuba8 | 0.001803 |
|  | GO:0005200 | structural constituent of cytoskeleton | 9 | Sptbn1,Tubb4b,Tln1,Tubb1,Tuba1a,Tubb2a,Tubb6,Tuba4a,Tuba8 | 0.006681 |
|  | GO:0044325 | ion channel binding | 6 | Flna,Diaph1,Hsp90ab1,Ywhae,Ywhaq,Hsp90aa1 | 0.010418 |
|  | GO:0003779 | actin binding | 10 | Actn4,Sptbn1,Parvb,Inf2,Diaph1,Cfl1,Ywhag,Pfn1,Twf1,Capzb | 0.014864 |
|  | GO:0017137 | Rab GTPase binding | 4 | Arhgap1,Sytl4,Unc13d,Dnm1l | 0.016823 |
|  | GO:0004175 | endopeptidase activity | 9 | Psma2,Psma1,Psma3,Psma4,Psmb8,Psmb3,Psmb2,Psma6,Psmb10 | 0.028773 |
|  | GO:0008536 | Ran GTPase binding | 3 | Ipo5,Ipo7,Kpnb1 | 0.046946 |
|  | GO:0032564 | dATP binding | 3 | Hsp90ab1,St13,Hsp90aa1 | 0.046946 |
|  | GO:0005096 | GTPase activator activity | 3 | Rasa3,Arhgap1,Arhgap45 | 0.046946 |
