# Supplementary material for: Immune Responses to Multi-Frequencies of 1.5 GHz and 4.3 GHz Microwave Exposure in Rats: Transcriptomic and Proteomic Analysis
Source: Int J Mol Sci. 2022 Jun 22;23(13):6949. doi: 10.3390/ijms23136949 (PMC9266614; doi:10.3390/ijms23136949)
Supplement: Supplementary file 1 [file ijms-23-06949-s001.zip › Supplementary Table S4.docx]

**Supplementary Table S4 GO analysis of DEPs between LC10 exposure and Sham exposure in spleen**

| **GO** | **ID** | **Categories** | **Number of DEPs** | **Name of proteins** | **P value** |
| --- | --- | --- | --- | --- | --- |
| BP | GO:0006782 | protoporphyrinogen IX biosynthetic process | 4 | Urod,Alas2,Alad,Cpox | 3.93E-06 |
|  | GO:0006783 | heme biosynthetic process | 4 | Fech,Alad,Cpox,Uros | 1.90E-05 |
|  | GO:0006270 | DNA replication initiation | 4 | Mcm5,Mcm6,Mcm2,Mcm7 | 5.49E-05 |
|  | GO:0070541 | response to platinum ion | 3 | Fech,Alad,Uros | 0.000345 |
|  | GO:0051597 | response to methylmercury | 3 | Fech,Alad,Cpox | 0.000835 |
|  | GO:0098586 | cellular response to virus | 2 | Fmr1,Adar | 0.002013 |
|  | GO:0042168 | heme metabolic process | 2 | Fech,Hpx | 0.002013 |
|  | GO:0045086 | positive regulation of interleukin-2 biosynthetic process | 2 | Stat5b,Glmn | 0.002013 |
|  | GO:0006268 | DNA unwinding involved in DNA replication | 3 | Mcm6,Mcm2,Mcm7 | 0.002733 |
|  | GO:0050871 | positive regulation of B cell activation | 3 | Igg-2a,Igh-1a,0 | 0.002733 |
|  | GO:0042742 | defense response to bacterium | 5 | Stab1,Igg-2a,Igh-1a,0,Irf8 | 0.003644 |
|  | GO:0006351 | transcription, DNA-templated | 3 | Ash2l,Polr2e,Col4a2 | 0.00423 |
|  | GO:0017085 | response to insecticide | 2 | Fech,Cpox | 0.005861 |
|  | GO:0045668 | negative regulation of osteoblast differentiation | 2 | Limd1,Cdk6 | 0.005861 |
|  | GO:0002092 | positive regulation of receptor internalization | 2 | Fmr1,Arrb1 | 0.005861 |
|  | GO:1902430 | negative regulation of amyloid-beta formation | 2 | Gga3,Apoe | 0.005861 |
|  | GO:0071353 | cellular response to interleukin-4 | 3 | Mrc1,Mcm2,Alad | 0.006138 |
|  | GO:0006910 | phagocytosis, recognition | 3 | Igg-2a,Igh-1a,0 | 0.006138 |
|  | GO:0046685 | response to arsenic-containing substance | 3 | Fech,Alad,Cpox | 0.006138 |
|  | GO:0006958 | complement activation, classical pathway | 3 | Igg-2a,Igh-1a,0 | 0.011284 |
|  | GO:0010288 | response to lead ion | 3 | Fech,Alad,Cpox | 0.018312 |
|  | GO:0090090 | negative regulation of canonical Wnt signaling pathway | 3 | Apoe,Tle1,Limd1 | 0.018312 |
|  | GO:0032715 | negative regulation of interleukin-6 production | 2 | Nlrx1,Arrb1 | 0.018404 |
|  | GO:2000637 | positive regulation of gene silencing by miRNA | 2 | Fmr1,Limd1 | 0.018404 |
|  | GO:0030195 | negative regulation of blood coagulation | 2 | Kng1,Map1 | 0.018404 |
|  | GO:0051258 | protein polymerization | 2 | Vtn,Fga | 0.018404 |
|  | GO:0006911 | phagocytosis, engulfment | 3 | Igg-2a,Igh-1a,0 | 0.022553 |
|  | GO:0098869 | cellular oxidant detoxification | 2 | Hba-a2,Hba-a3 | 0.026799 |
|  | GO:0010035 | response to inorganic substance | 2 | Alad,Cpox | 0.026799 |
|  | GO:0030212 | hyaluronan metabolic process | 2 | Itih3,Itih4 | 0.026799 |
|  | GO:0033077 | T cell differentiation in thymus | 2 | Stat5b,Cdk6 | 0.026799 |
|  | GO:0010039 | response to iron ion | 2 | Alad,Cpox | 0.026799 |
|  | GO:0010212 | response to ionizing radiation | 2 | Alad,Babam1 | 0.026799 |
|  | GO:2001235 | positive regulation of apoptotic signaling pathway | 2 | Eef1e1,Apaf1 | 0.026799 |
|  | GO:0006956 | complement activation | 2 | Cfb,C4b | 0.026799 |
|  | GO:0006879 | cellular iron ion homeostasis | 3 | Alas2,Tfrc,Hpx | 0.027282 |
|  | GO:0042744 | hydrogen peroxide catabolic process | 3 | Hba-a2,Pxdn,Hba-a3 | 0.027282 |
|  | GO:0042632 | cholesterol homeostasis | 3 | Apoe,Sec24a,Npc2 | 0.032498 |
|  | GO:0045931 | positive regulation of mitotic cell cycle | 2 | Stat5b,Asns | 0.036425 |
|  | GO:0045739 | positive regulation of DNA repair | 2 | Pcna,Babam1 | 0.036425 |
|  | GO:0008277 | regulation of G protein-coupled receptor signaling pathway | 2 | Rgs18,Arrb1 | 0.036425 |
|  | GO:0010038 | response to metal ion | 2 | Fech,Alad | 0.036425 |
|  | GO:0030900 | forebrain development | 2 | Dnajb1,Apaf1 | 0.036425 |
|  | GO:0071364 | cellular response to epidermal growth factor stimulus | 3 | Plcg1,Mcm7,Baiap2 | 0.038195 |
|  | GO:0050853 | B cell receptor signaling pathway | 3 | Igg-2a,Igh-1a,0 | 0.038195 |
|  | GO:1900369 | negative regulation of RNA interference | 1 | Adar | 0.045042 |
|  | GO:0006272 | leading strand elongation | 1 | Pcna | 0.045042 |
|  | GO:0045714 | regulation of low-density lipoprotein particle receptor biosynthetic process | 1 | Sec24a | 0.045042 |
|  | GO:0046543 | development of secondary female sexual characteristics | 1 | Stat5b | 0.045042 |
|  | GO:1903259 | exon-exon junction complex disassembly | 1 | Pym1 | 0.045042 |
|  | GO:0046683 | response to organophosphorus | 1 | Tyms | 0.045042 |
|  | GO:0016071 | mRNA metabolic process | 1 | Tbrg4 | 0.045042 |
|  | GO:2000822 | regulation of behavioral fear response | 1 | Apoe | 0.045042 |
|  | GO:0046544 | development of secondary male sexual characteristics | 1 | Stat5b | 0.045042 |
|  | GO:0032793 | positive regulation of CREB transcription factor activity | 1 | Prkd2 | 0.045042 |
|  | GO:0043697 | cell dedifferentiation | 1 | Cdk6 | 0.045042 |
|  | GO:0042448 | progesterone metabolic process | 1 | Stat5b | 0.045042 |
|  | GO:0070669 | response to interleukin-2 | 1 | Stat5b | 0.045042 |
|  | GO:0032354 | response to follicle-stimulating hormone | 1 | Asns | 0.045042 |
|  | GO:0090344 | negative regulation of cell aging | 1 | 43895 | 0.045042 |
|  | GO:0001779 | natural killer cell differentiation | 1 | Stat5b | 0.045042 |
|  | GO:1901628 | positive regulation of postsynaptic membrane organization | 1 | Apoe | 0.045042 |
|  | GO:0045616 | regulation of keratinocyte differentiation | 1 | Rock2 | 0.045042 |
|  | GO:0071048 | nuclear retention of unspliced pre-mRNA at the site of transcription | 1 | Exosc10 | 0.045042 |
|  | GO:0042541 | hemoglobin biosynthetic process | 1 | Alas2 | 0.045042 |
|  | GO:0035331 | negative regulation of hippo signaling | 1 | Limd1 | 0.045042 |
|  | GO:1905855 | positive regulation of heparan sulfate binding | 1 | Apoe | 0.045042 |
|  | GO:0045647 | negative regulation of erythrocyte differentiation | 1 | Stat5b | 0.045042 |
|  | GO:0099564 | modification of synaptic structure, modulating synaptic transmission | 1 | Baiap2 | 0.045042 |
|  | GO:0042158 | lipoprotein biosynthetic process | 1 | Apoe | 0.045042 |
|  | GO:0046501 | protoporphyrinogen IX metabolic process | 1 | Fech | 0.045042 |
|  | GO:1902373 | negative regulation of mRNA catabolic process | 1 | Fmr1 | 0.045042 |
|  | GO:1902510 | regulation of apoptotic DNA fragmentation | 1 | Apaf1 | 0.045042 |
|  | GO:0032077 | positive regulation of deoxyribonuclease activity | 1 | Pcna | 0.045042 |
|  | GO:0098908 | regulation of neuronal action potential | 1 | Fmr1 | 0.045042 |
|  | GO:0046078 | dUMP metabolic process | 1 | Tyms | 0.045042 |
|  | GO:0019088 | immortalization of host cell by virus | 1 | Tyms | 0.045042 |
|  | GO:0008156 | negative regulation of DNA replication | 1 | Pds5a | 0.045042 |
|  | GO:0002925 | positive regulation of humoral immune response mediated by circulating immunoglobulin | 1 | Hpx | 0.045042 |
|  | GO:0044830 | modulation by host of viral RNA genome replication | 1 | Fmr1 | 0.045042 |
|  | GO:2001287 | negative regulation of caveolin-mediated endocytosis | 1 | Unc119 | 0.045042 |
|  | GO:0048213 | Golgi vesicle prefusion complex stabilization | 1 | Cog4 | 0.045042 |
|  | GO:0071466 | cellular response to xenobiotic stimulus | 1 | Mcm7 | 0.045042 |
|  | GO:1901630 | negative regulation of presynaptic membrane organization | 1 | Apoe | 0.045042 |
|  | GO:1900272 | negative regulation of long-term synaptic potentiation | 1 | Apoe | 0.045042 |
|  | GO:0010729 | positive regulation of hydrogen peroxide biosynthetic process | 1 | Mtco2 | 0.045042 |
|  | GO:0019218 | regulation of steroid metabolic process | 1 | Stat5b | 0.045042 |
|  | GO:0006780 | uroporphyrinogen III biosynthetic process | 1 | Uros | 0.045042 |
|  | GO:1901727 | positive regulation of histone deacetylase activity | 1 | Prkd2 | 0.045042 |
|  | GO:0007602 | phototransduction | 1 | Arrb1 | 0.045042 |
|  | GO:0071831 | intermediate-density lipoprotein particle clearance | 1 | Apoe | 0.045042 |
|  | GO:1905890 | regulation of cellular response to very-low-density lipoprotein particle stimulus | 1 | Apoe | 0.045042 |
|  | GO:0044237 | cellular metabolic process | 1 | Eif2b4 | 0.045042 |
|  | GO:0032462 | regulation of protein homooligomerization | 1 | Apoe | 0.045042 |
|  | GO:0060574 | intestinal epithelial cell maturation | 1 | Tyms | 0.045042 |
|  | GO:0010544 | negative regulation of platelet activation | 1 | Apoe | 0.045042 |
|  | GO:1902167 | positive regulation of intrinsic apoptotic signaling pathway in response to DNA damage by p53 class mediator | 1 | Rpl26 | 0.045042 |
|  | GO:0032805 | positive regulation of low-density lipoprotein particle receptor catabolic process | 1 | Apoe | 0.045042 |
|  | GO:0070981 | L-asparagine biosynthetic process | 1 | Asns | 0.045042 |
|  | GO:0006231 | dTMP biosynthetic process | 1 | Tyms | 0.045042 |
|  | GO:0034447 | very-low-density lipoprotein particle clearance | 1 | Apoe | 0.045042 |
|  | GO:1903205 | regulation of hydrogen peroxide-induced cell death | 1 | Stk26 | 0.045042 |
|  | GO:0032717 | negative regulation of interleukin-8 production | 1 | Arrb1 | 0.045042 |
|  | GO:0010266 | response to vitamin B1 | 1 | Alad | 0.045042 |
|  | GO:0090240 | positive regulation of histone H4 acetylation | 1 | Arrb1 | 0.045042 |
|  | GO:1903002 | positive regulation of lipid transport across blood-brain barrier | 1 | Apoe | 0.045042 |
|  | GO:1902036 | regulation of hematopoietic stem cell differentiation | 1 | Cdk6 | 0.045042 |
|  | GO:0006529 | asparagine biosynthetic process | 1 | Asns | 0.045042 |
|  | GO:0010825 | positive regulation of centrosome duplication | 1 | Rock2 | 0.045042 |
|  | GO:1905906 | regulation of amyloid fibril formation | 1 | Apoe | 0.045042 |
|  | GO:0032735 | positive regulation of interleukin-12 production | 1 | Irf8 | 0.045042 |
|  | GO:0030856 | regulation of epithelial cell differentiation | 1 | Stat5b | 0.045042 |
|  | GO:0006382 | adenosine to inosine editing | 1 | Adar | 0.045042 |
|  | GO:0002566 | somatic diversification of immune receptors via somatic mutation | 1 | Adar | 0.045042 |
|  | GO:0032825 | positive regulation of natural killer cell differentiation | 1 | Stat5b | 0.045042 |
|  | GO:1904803 | regulation of translation involved in cellular response to UV | 1 | Rpl26 | 0.045042 |
|  | GO:0042982 | amyloid precursor protein metabolic process | 1 | Apoe | 0.045042 |
|  | GO:2001033 | negative regulation of double-strand break repair via nonhomologous end joining | 1 | Nudt16l1 | 0.045042 |
|  | GO:0072377 | blood coagulation, common pathway | 1 | Fga | 0.045042 |
|  | GO:0033014 | tetrapyrrole biosynthetic process | 1 | Hmbs | 0.045042 |
|  | GO:1905775 | negative regulation of DNA helicase activity | 1 | Mcm2 | 0.045042 |
|  | GO:0044528 | regulation of mitochondrial mRNA stability | 1 | Tbrg4 | 0.045042 |
|  | GO:0007263 | nitric oxide mediated signal transduction | 1 | Apoe | 0.045042 |
|  | GO:0097114 | NMDA glutamate receptor clustering | 1 | Apoe | 0.045042 |
|  | GO:1902952 | positive regulation of dendritic spine maintenance | 1 | Apoe | 0.045042 |
|  | GO:0097531 | mast cell migration | 1 | Stat5b | 0.045042 |
|  | GO:1905860 | positive regulation of heparan sulfate proteoglycan binding | 1 | Apoe | 0.045042 |
|  | GO:0019860 | uracil metabolic process | 1 | Tyms | 0.045042 |
|  | GO:0089700 | protein kinase D signaling | 1 | Prkd2 | 0.045042 |
|  | GO:0002176 | male germ cell proliferation | 1 | Eif2s2 | 0.045042 |
|  | GO:1902164 | positive regulation of DNA damage response, signal transduction by p53 class mediator resulting in transcription of p21 class mediator | 1 | Rpl26 | 0.045042 |
|  | GO:0018160 | peptidyl-pyrromethane cofactor linkage | 1 | Hmbs | 0.045042 |
|  | GO:0097305 | response to alcohol | 1 | H6pd | 0.045042 |
|  | GO:0019353 | protoporphyrinogen IX biosynthetic process from glutamate | 1 | Cpox | 0.045042 |
|  | GO:2000773 | negative regulation of cellular senescence | 1 | Cdk6 | 0.045042 |
|  | GO:0090615 | mitochondrial mRNA processing | 1 | Tbrg4 | 0.045042 |
|  | GO:0070672 | response to interleukin-15 | 1 | Stat5b | 0.045042 |
|  | GO:0003323 | type B pancreatic cell development | 1 | Cdk6 | 0.045042 |
|  | GO:0008612 | peptidyl-lysine modification to peptidyl-hypusine | 1 | Dohh | 0.045042 |
|  | GO:0097113 | AMPA glutamate receptor clustering | 1 | Apoe | 0.045042 |
|  | GO:0046081 | dUTP catabolic process | 1 | Dut | 0.045042 |
|  | GO:0002031 | G protein-coupled receptor internalization | 1 | Arrb1 | 0.045042 |
|  | GO:0150033 | negative regulation of protein localization to lysosome | 1 | Rock2 | 0.045042 |
|  | GO:1901254 | positive regulation of intracellular transport of viral material | 1 | Fmr1 | 0.045042 |
|  | GO:0050714 | positive regulation of protein secretion | 2 | Sec24a,Fga | 0.047156 |
|  | GO:0031648 | protein destabilization | 2 | Gga3,Derl1 | 0.047156 |
|  | GO:0070374 | positive regulation of ERK1 and ERK2 cascade | 4 | Apoe,Arrb1,Prkd2,Fga | 0.048141 |
| CC | GO:0042555 | MCM complex | 4 | Mcm5,Mcm6,Mcm2 | 6.00E-05 |
|  | GO:0042571 | immunoglobulin complex, circulating | 3 | Igg-2a,Igh-1a,0 | 0.002915 |
|  | GO:0000785 | chromatin | 4 | Pds5a,Mcm2,Pcna,Arrb1 | 0.016459 |
|  | GO:0031838 | haptoglobin-hemoglobin complex | 2 | Hba-a2,Hba-a3 | 0.019201 |
|  | GO:0009897 | external side of plasma membrane | 6 | Ms4a1,Tfrc,Igg-2a,Igh-1a,0,Fga | 0.019293 |
|  | GO:0031012 | extracellular matrix | 4 | Apoe,Mmrn1,Col6a2,Col4a2 | 0.025593 |
|  | GO:0099524 | postsynaptic cytosol | 2 | Dnajb1,Baiap2 | 0.037951 |
|  | GO:0001940 | male pronucleus | 1 | Cbx1 | 0.046046 |
|  | GO:0033118 | esterosome membrane | 1 | Gga3 | 0.046046 |
|  | GO:0048500 | signal recognition particle | 1 | Derl1 | 0.046046 |
|  | GO:0061845 | neuron projection branch point | 1 | Baiap2 | 0.046046 |
|  | GO:0070557 | PCNA-p21 complex | 1 | Pcna | 0.046046 |
|  | GO:0005796 | Golgi lumen | 1 | Vtn | 0.046046 |
|  | GO:0043293 | apoptosome | 1 | Apaf1 | 0.046046 |
|  | GO:0043596 | nuclear replication fork | 1 | Pcna | 0.046046 |
|  | GO:0019034 | viral replication complex | 1 | Fmr1 | 0.046046 |
|  | GO:0048237 | rough endoplasmic reticulum lumen | 1 | Vtn | 0.046046 |
|  | GO:0005664 | nuclear origin of replication recognition complex | 1 | Mcm2 | 0.046046 |
|  | GO:0097132 | cyclin D2-CDK6 complex | 1 | Cdk6 | 0.046046 |
|  | GO:0043626 | PCNA complex | 1 | Pcna | 0.046046 |
|  | GO:0045277 | respiratory chain complex IV | 1 | Mtco2 | 0.046046 |
|  | GO:0061846 | dendritic spine cytoplasm | 1 | Baiap2 | 0.046046 |
|  | GO:0036502 | Derlin-1-VIMP complex | 1 | Derl1 | 0.046046 |
|  | GO:0000308 | cytoplasmic cyclin-dependent protein kinase holoenzyme complex | 1 | Ccny | 0.046046 |
|  | GO:0035327 | transcriptionally active chromatin | 2 | Hist1h1c,Exosc10 | 0.049098 |
|  | GO:0005833 | hemoglobin complex | 2 | Hba-a2,Hba-a3 | 0.049098 |
|  | GO:0005743 | mitochondrial inner membrane | 5 | Alas2,Mtco2,Tyms,Cpox,Abcb10 | 0.049334 |
| MF | GO:0048037 | cofactor binding | 4 | Tyms,Asns,Dohh,Uros | 0.000244 |
|  | GO:0034987 | immunoglobulin receptor binding | 3 | Igg-2a,Igh-1a,0 | 0.006247 |
|  | GO:0003823 | antigen binding | 3 | Igg-2a,Igh-1a,0 | 0.008631 |
|  | GO:0004386 | helicase activity | 3 | Mcm5,Mcm2,Ddx47 | 0.011479 |
|  | GO:0003688 | DNA replication origin binding | 2 | Mcm5,Mcm2 | 0.011515 |
|  | GO:0031434 | mitogen-activated protein kinase kinase binding | 2 | Ksr1,Arrb1 | 0.011515 |
|  | GO:0043177 | organic acid binding | 2 | Hba-a2,Hba-a3 | 0.018625 |
|  | GO:1990825 | sequence-specific mRNA binding | 2 | Fmr1,Tyms | 0.036848 |
|  | GO:0030246 | carbohydrate binding | 4 | Nomo1,H6pd,Mrc2,Prps2, | 0.04424 |
|  | GO:0001221 | transcription cofactor binding | 1 | Baiap2 | 0.045323 |
|  | GO:0015232 | heme transporter activity | 1 | Hpx | 0.045323 |
|  | GO:0045309 | protein phosphorylated amino acid binding | 1 | Arrb1 | 0.045323 |
|  | GO:0032556 | pyrimidine deoxyribonucleotide binding | 1 | Dut | 0.045323 |
|  | GO:0005171 | hepatocyte growth factor receptor binding | 1 | Glmn | 0.045323 |
|  | GO:0004418 | hydroxymethylbilane synthase activity | 1 | Hmbs | 0.045323 |
|  | GO:0017171 | serine hydrolase activity | 1 | Nceh1 | 0.045323 |
|  | GO:0019135 | deoxyhypusine monooxygenase activity | 1 | Dohh | 0.045323 |
|  | GO:0033570 | transferrin transmembrane transporter activity | 1 | Tfrc | 0.045323 |
|  | GO:0004613 | phosphoenolpyruvate carboxykinase (GTP) activity | 1 | Pck2 | 0.045323 |
|  | GO:0030547 | receptor inhibitor activity | 1 | Dut | 0.045323 |
|  | GO:0031691 | alpha-1A adrenergic receptor binding | 1 | Arrb1 | 0.045323 |
|  | GO:0046911 | metal chelating activity | 1 | Apoe | 0.045323 |
|  | GO:0004852 | uroporphyrinogen-III synthase activity | 1 | Uros | 0.045323 |
|  | GO:0098770 | FBXO family protein binding | 1 | Cdk6 | 0.045323 |
|  | GO:0004998 | transferrin receptor activity | 1 | Tfrc | 0.045323 |
|  | GO:0030151 | molybdenum ion binding | 1 | Mar-2 | 0.045323 |
|  | GO:0005542 | folic acid binding | 1 | Tyms | 0.045323 |
|  | GO:1904854 | proteasome core complex binding | 1 | Alad | 0.045323 |
|  | GO:0003870 | 5-aminolevulinate synthase activity | 1 | Alas2 | 0.045323 |
|  | GO:0030306 | ADP-ribosylation factor binding | 1 | Gga3 | 0.045323 |
|  | GO:0000701 | purine-specific mismatch base pair DNA N-glycosylase activity | 1 | Pcna | 0.045323 |
|  | GO:0003726 | double-stranded RNA adenosine deaminase activity | 1 | Adar | 0.045323 |
|  | GO:0055105 | ubiquitin-protein transferase inhibitor activity | 1 | Glmn | 0.045323 |
|  | GO:0016538 | cyclin-dependent protein serine/threonine kinase regulator activity | 1 | Ccny | 0.045323 |
|  | GO:0070326 | very-low-density lipoprotein particle receptor binding | 1 | Apoe | 0.045323 |
|  | GO:0004799 | thymidylate synthase activity | 1 | Tyms | 0.045323 |
|  | GO:0031701 | angiotensin receptor binding | 1 | Arrb1 | 0.045323 |
|  | GO:0004045 | aminoacyl-tRNA hydrolase activity | 1 | Ptrh2 | 0.045323 |
|  | GO:1990188 | euchromatin binding | 1 | Ash2l | 0.045323 |
|  | GO:0004109 | coproporphyrinogen oxidase activity | 1 | Cpox | 0.045323 |
|  | GO:0030337 | DNA polymerase processivity factor activity | 1 | Pcna | 0.045323 |
|  | GO:0031692 | alpha-1B adrenergic receptor binding | 1 | Arrb1 | 0.045323 |
|  | GO:0002151 | G-quadruplex RNA binding | 1 | Fmr1 | 0.045323 |
|  | GO:0032139 | dinucleotide insertion or deletion binding | 1 | Pcna | 0.045323 |
|  | GO:0004655 | porphobilinogen synthase activity | 1 | Alad | 0.045323 |
|  | GO:0072518 | Rho-dependent protein serine/threonine kinase activity | 1 | Rock2 | 0.045323 |
|  | GO:0031896 | V2 vasopressin receptor binding | 1 | Arrb1 | 0.045323 |
|  | GO:0004325 | ferrochelatase activity | 1 | Fech | 0.045323 |
|  | GO:0004853 | uroporphyrinogen decarboxylase activity | 1 | Urod | 0.045323 |
|  | GO:0004170 | dUTP diphosphatase activity | 1 | Dut | 0.045323 |
|  | GO:0050290 | sphingomyelin phosphodiesterase D activity | 1 | Smpd4 | 0.045323 |
|  | GO:0004066 | asparagine synthase (glutamine-hydrolyzing) activity | 1 | Asns | 0.045323 |
|  | GO:0005344 | oxygen carrier activity | 2 | Hba-a2,Hba-a3 | 0.047695 |
|  | GO:0017048 | Rho GTPase binding | 2 | Myo9b,Rock2 | 0.047695 |
|  | GO:0008266 | poly(U) RNA binding | 2 | Fmr1,Pabpc4 | 0.047695 |
