# Supplementary material for: Immune Responses to Multi-Frequencies of 1.5 GHz and 4.3 GHz Microwave Exposure in Rats: Transcriptomic and Proteomic Analysis
Source: Int J Mol Sci. 2022 Jun 22;23(13):6949. doi: 10.3390/ijms23136949 (PMC9266614; doi:10.3390/ijms23136949)
Supplement: Supplementary file 1 [file ijms-23-06949-s001.zip › Supplementary Table S5.docx]

**Supplementary Table S5 The sequence of primers for real-time RT-PCR**

| **Genes** | **Primers (5’ to 3’)** |
| --- | --- |
| **Cxcl1** | Sense: ACCGAAGTCATAGCCACACT  Anti-sense: CGCCATCGGTGCAATCTATC |
| **Cth** | Sense: CTACCCTCGGGATCAGTGAC  Anti-sense: CCGGCTTTGACTCGAACTTT |
| **Dnaja1** | Sense: AGGACTGGAGCCAGGAGATA  Anti-sense: TCTGACCTGGATGAGAGGTG |
| **Fech** | Sense:mCTCCTCATCCAGTGCTTTGC  Anti-sense: ATAACCCAGCTGCTCCATGA |
| **Hsph1** | Sense: GCGCTAAGGTCAAGGAACTG  Anti-sense: CCCTTCTCATTAGGGTGGCA |
| **Hsp90aa1** | Sense: CCTTCATGGAGGCTTTGCAG  Anti-sense: CCCATTGGTTCACCTGTGTC |
| **Mpp4** | Sense: AAAGTGCTGTGCCACATACC  Anti-sense: CCTTCGTCAAGGACTGCAAG |
| **Mx2** | Sense: GAGGAGGAGAGGAAGCATGG  Anti-sense: TCTGCAGCTTCTCAGCAAAC |
| **Ppp1cb** | Sense: TGTCATGGAGGACTGTCACC  Anti-sense: TGGGTCGGACCACAGTAAAT |
| **Prok2** | Sense: CATCACCGGGGCTTGCGACA  Anti-sense: TCCGAGTCAGGGGGTGGCAG |
| **Rpl7a** | Sense: CAGGAAGACATGCACCACTG  Anti-sense: CGGCGGATCTCGTCATATCT |
| **Samhd1** | Sense: AGCCACAGGATGGTGACATT  Anti-sense: AGGTGGCCTCTGATTCTGTT |
| **Serpine1** | Sense: AGTCTTTCCGACCAAGAGCA  Anti-sense: GGGCTGAGACTAGAATGGCT |
| **Tgfb1** | Sense: TGCTTCAGCTCCACAGAGAA  Anti-sense: TCCAGGCTCCAAATGTAGGG |
| **GAPDH** | Sense: CAAGGCTGAGAATGGGAAGC  Anti-sense: GAAGACGCCAGTAGACTCCA |
